# Comparative genome analysis of *Vagococcus fluvialis* reveals abundance of mobile genetic elements in sponge-isolated strains

**DOI:** 10.1186/s12864-022-08842-9

**Published:** 2022-08-25

**Authors:** Ana Rodriguez Jimenez, Nadège Guiglielmoni, Lise Goetghebuer, Etienne Dechamps, Isabelle F. George, Jean-François Flot

**Affiliations:** 1 Ecology of Aquatic Systems, Université libre de Bruxelles (ULB), Brussels, Belgium; 2 Evolutionary Biology and Ecology, Université libre de Bruxelles (ULB), Brussels, Belgium; 3 Marine Biology, Université libre de Bruxelles (ULB), Brussels, Belgium; 4 Interuniversity Institute of Bioinformatics in Brussels – (IB)², Brussels, Belgium

**Keywords:** *Vagococcus fluvialis*, Genome assembly, Hybrid assembly, Sponge bacteria, Mobile genetic elements

## Abstract

**Background:**

*Vagococcus fluvialis* is a species of lactic acid bacteria found both free-living in river and seawater and associated to hosts, such as marine sponges. This species has been greatly understudied, with no complete genome assembly available to date, which is essential for the characterisation of the mobilome.

**Results:**

We sequenced and assembled de novo the complete genome sequences of five *V. fluvialis* isolates recovered from marine sponges. Pangenome analysis of the *V. fluvialis* species (total of 17 genomes) showed a high intraspecific diversity, with 45.5% of orthologous genes found to be strain specific. Despite this diversity, analyses of gene functions clustered all *V. fluvialis* species together and separated them from other sequenced *Vagococcus* species. *V. fluvialis* strains from different habitats were highly similar in terms of functional diversity but the sponge-isolated strains were enriched in several functions related to the marine environment. Furthermore, sponge-isolated strains carried a significantly higher number of mobile genetic elements (MGEs) compared to previously sequenced *V. fluvialis* strains from other environments. Sponge-isolated strains carried up to 4 circular plasmids each, including a 48-kb conjugative plasmid. Three of the five strains carried an additional circular extrachromosomal sequence, assumed to be an excised prophage as it contained mainly viral genes and lacked plasmid replication genes. Insertion sequences (ISs) were up to five times more abundant in the genomes of sponge-isolated strains compared to the others, including several IS families found exclusively in these genomes.

**Conclusions:**

Our findings highlight the dynamics and plasticity of the *V. fluvialis* genome. The abundance of mobile genetic elements in the genomes of sponge-isolated *V. fluvialis* strains suggests that the mobilome might be key to understanding the genomic signatures of symbiosis in bacteria.

**Supplementary Information:**

The online version contains supplementary material available at 10.1186/s12864-022-08842-9.

## Background

Marine sponges harbour highly diverse, specific and dynamic microbial communities [1]. The filter-feeding activity of sponges constantly exposes their microbiome to planktonic microorganisms [2]. The high viral abundance in oceans, together with the phagocytic feeding mode of sponges, result in a prevalence of horizontal gene transfer (HGT) in the sponge environment [3, 4]. Such high levels of genetic exchange within the sponge microbiome is expected to shape its evolution and its capacity to adapt to environmental changes [5]. However, studies of sponge-associated microbionts have so far been hampered by a dearth of completely assembled genomes, restricting comparisons to gene-coding sequences, and hampering comparison of mobile genetic elements (such as phages, plasmids, and transposable elements). As a result, a comparison of the mobilome of free-living vs. sponge-associated bacteria is still lacking.

One particularly interesting taxon for such comparison is *Vagococcus fluvialis*, a species of Gram-positive, motile coccoid bacteria belonging to the order Lactobacillales (lactic acid bacteria) that is found in a variety of environments including mammals, fish, birds, rivers, seawater, and sponges. The genus *Vagococcus* is phylogenetically and phenotypically close to the genera *Enterococcus* and *Lactococcus*. The species *Vagococcus fluvialis* was proposed in 1989 and accepted in 1990, when it was first classified as ‘motile group N streptococci’ [6, 7]. All known *V. fluvialis* strains are facultatively anaerobic, chemoorganotrophic, and fermentative. A characteristics of this species is its unique pattern of membrane lipids and fatty acids, containing rare compounds and a specific lipid named d-alanylcardiolipin [8]. Probiotic and immunomodulatory properties of a *V. fluvialis* strain in fish have been reported [9, 10]. Isolates affiliated to this species have also associated to human infections, albeit rarely [6].

Although lactic acid bacteria are widely studied, in particular for their role in fermented food production, the *Vagococcus* genus is as been relatively neglected so far [11, 12]. Only few studies have been carried out to characterize genome sequences from this genus. To date, only three *Vagococcus* species have had their genome completely assembled. Although *V. fluvialis* was the first species described of this genus, its complete genome sequence has not been assembled. To date, thirteen, fragmented, genome sequences of this species are available, most of which were obtained very recently [13].

Previously, we isolated bacteria associated to the marine sponges *Hymeniacidon perlevis* and *Halichondria panicea*, sampled in the intertidal zone at Wimereux, France [14]. In the bacterial collection retrieved from the sponges’ tissue, five strains were affiliated to the species *V. fluvialis* according to their 16S rRNA gene. In the present study, we performed whole-genome sequencing of these five *V. fluvialis* isolates (12B2, 25B2, 35B2, 36B2 and 110B2). We used long reads from Oxford Nanopore Technologies combined with high-accuracy Illumina NovaSeq reads to assemble de novo the complete genomes. This allowed us to perform a comparative genome analysis with all publicly available *V. fluvialis* genomes.

## Results

### Assembly and general characteristics of the *V. fluvialis* genomes

The complete genomes of the sponge-isolated *V. fluvialis* strains consisted of a circular chromosome and 1 to 4 circular extrachromosomal sequences. The five genomes were taxonomically classified as *Vagococcus fluvialis* by GTDB-Tk. The K-mer completeness ranged from 99.69 to 100% (Table 1). The correctness and circular nature of all circular contigs were confirmed by manual inspection of read mapping. The newly assembled genomes had a BUSCO completeness of 99.5% and CheckM completeness of 99.45%, equivalent to those of the previously assembled genomes.

**Table 1:** General sequencing, assembly, and genomic characteristics of the *Vagococcus fluvialis* genomes

Strain	Origin	Chromosome length (Mb)	Long-read coverage	Short-read coverage	BUSCO completeness	K-mer completeness	CheckM completeness (contamination)	CDSs	tRNA genes	rRNA genes (5S, 16S, 23S)	CRISPRs (Spacers, Cas)	GC %
12B2	Sponge	2.77	114x	316x	99.5%	99.77%	99.45% (0.83%)	2774	78	21 (7, 7, 7)	2 (2, 0)	32.7
35B2	Sponge	2.77	182x	198x	99.5%	99.69%	99.45% (0.83%)	2772	78	21 (7, 7, 7)	2 (2, 0)	32.7
110B2	Sponge	2.77	84x	250x	99.5%	100.00%	99.45% (0.83%)	2783	78	21 (7, 7, 7)	2 (2, 0)	32.7
25B2	Sponge	2.76	171x	280x	99.5%	99.93%	99.45% (0.55%)	2720	77	21 (7, 7, 7)	1 (1, 0)	32.6
36B2	Sponge	2.81	140x	278x	99.5%	99.95%	99.45% (0.28%)	2764	78	21 (7, 7, 7)	3 (3, 0)	32.6
DSM5731	Chicken	2.65	NA	553x	99.5%	NA	99.45% (0.83%)	2604	36	6 (4, 1, 1)	2 (2, 1)	32.4
NCDO2497	Chicken	2.65	NA	197x	99.5%	NA	99.45% (0.83%)	2610	42	4 (2, 1, 1)	1 (2, 1)	32.4
UFMGH6	Bovine	2.68	NA	182x	99.5%	NA	99.45% (0.83%)	2663	50	4 (2, 1, 1)	1 (1, 1)	32.4
UFMGH6B	Bovine	2.85	NA	38x	99.5%	NA	99.45% (3.04%)	2825	50	5 (3, 1, 1)	2 (32, 2)	33.6
UFMGH7	Bovine	2.99	NA	165x	99.5%	NA	99.45% (0.83%)	2937	52	4 (2, 1, 1)	1 (1, 1)	32.3
DIV0015	Aquarium water	2.88	NA	73x	99.5%	NA	99.45% (0.28%)	2779	22	3 (1, 1, 1)	0 (0, 0)	32.3
DIV0038b	Chicken	2.83	NA	56x	99.5%	NA	99.45% (0.28%)	2750	29	4 (2, 1, 1)	1 (1, 2)	32.3
DIV0068	Aquarium water	2.74	NA	124x	99.5%	NA	99.45% (0.97%)	2749	16	3 (1, 1, 1)	1 (1, 4)	32.4
DIV0098	Aquarium water	2.68	NA	59x	99.5%	NA	99.45% (0.41%)	2597	22	3 (1, 1, 1)	1 (36, 4)	32.3
DIV0648b	Turkey	3.13	NA	129x	99.5%	NA	99.45% (0.28%)	3048	28	3 (1, 1, 1)	1 (1, 0)	32.3
DIV0657d	Turkey	2.74	NA	53x	99.5%	NA	99.45% (0.97%)	2747	15	3 (1, 1, 1)	0 (0, 4)	32.4
MSG3302	Turtle	2.76	NA	159x	99.5%	NA	99.45% (0.83%)	2767	8	3 (1, 1, 1)	0 (0, 0)	32.3

The 17 *V. fluvialis* genomes (5 genomes newly sequenced in this study plus 12 genomes retrieved from the NCBI database) (Supplementary Table S1) had a size of 2.65 to 3.13 Mb and displayed between 97.3 and 100% average amino acid identity (AAI) (Supplementary Figure S1). The genomes of 12B2, 35B2 and 110B2 had 100% AAI. The two other sponge-isolated strain genomes were slightly different from the former, with AAIs of 98.0 and 97.7% for 36B2 and 25B2, respectively. Notably, the 16S rRNA gene sequences in the genomes of these five strains were identical, as previously observed using Sanger sequencing of PCR amplicons [14].

We found a significant difference in the number of tRNA and rRNA genes detected in the newly assembled genomes compared to the previously published, fragmented assemblies (21 to 72 contigs; Table 1). The number of tRNA genes in our new assemblies ranged from 77 to 78 but ranged from 8 to 50 (with an average of 31) in the previously published *V. fluvialis* genomes. We identified 21 rRNA genes, representing 7 identical complete rRNA operons in the 5 genomes sequenced in this study, while only 3 to 6 rRNA genes were present in the previously published genomes in incomplete *rRNA* operons (Table 1). We reassembled our genomes using only short reads to evaluate the biological or assembly-linked nature of the differences observed with the other genomes. These fragmented assemblies contained less than half of the number of tRNA and rRNA genes found in the corresponding complete assemblies (Supplementary Table S2).

### Comparative genome analysis of *Vagococcus fluvialis*

**Fig. 1:**
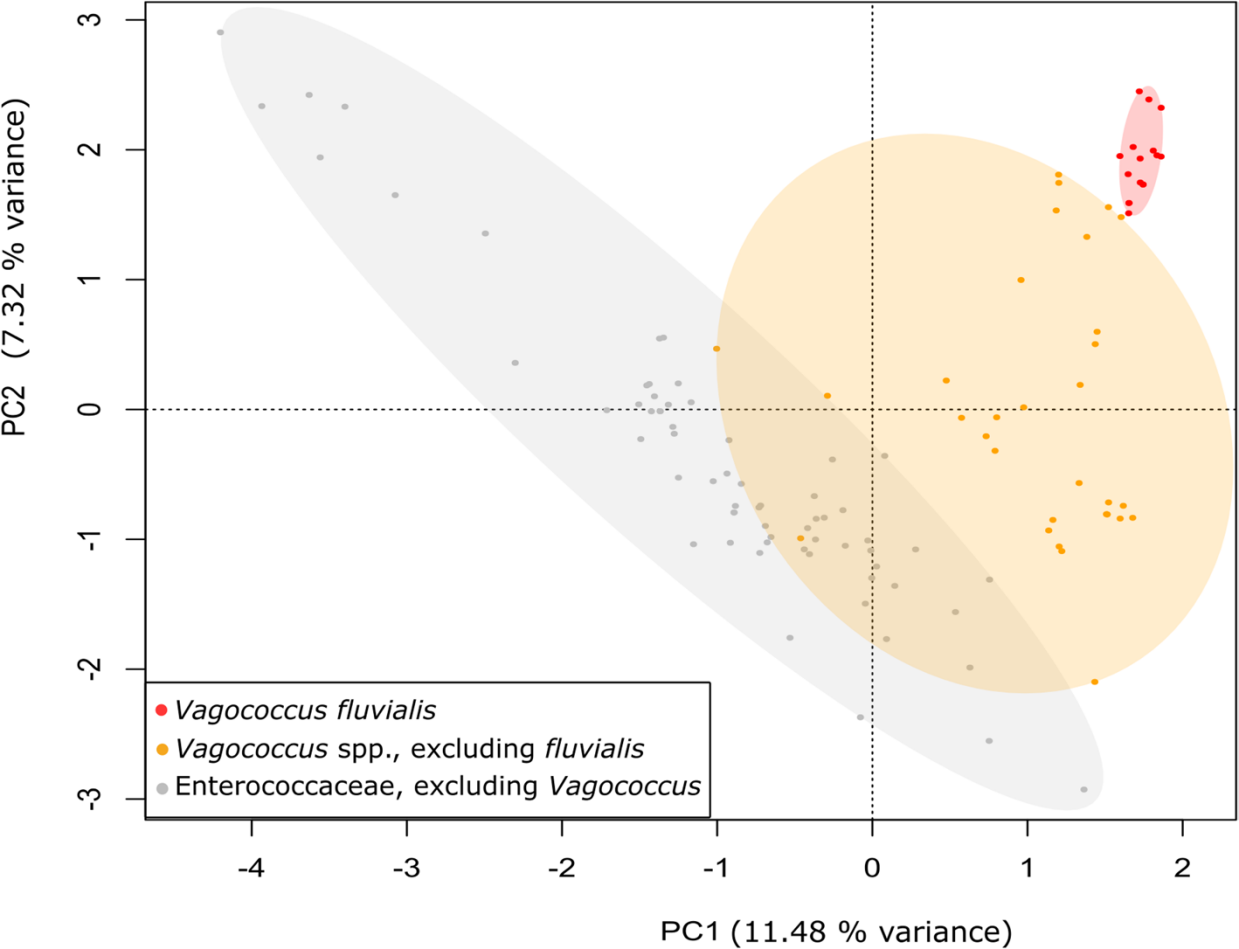
Principal Component Analysis (PCA) of Enterococcaceae KEGG Orthologous (KO) gene annotation. Color-coded transparent ellipsis group all points of each taxonomy group

The genomes of *Vagococcus fluvialis* formed a distinct functional cluster when compared to those of other *Vagococcu*s and more generally Enterococcaceae genomes (Fig. 1). *V. fluvialis* genomes were systematically enriched in the KEGG pathway categories of amino acid metabolism, xenobiotics biodegradation and metabolism, membrane transport, signal transduction and antimicrobial drug resistance (Supplementary Figure S2).

**Fig. 2:**
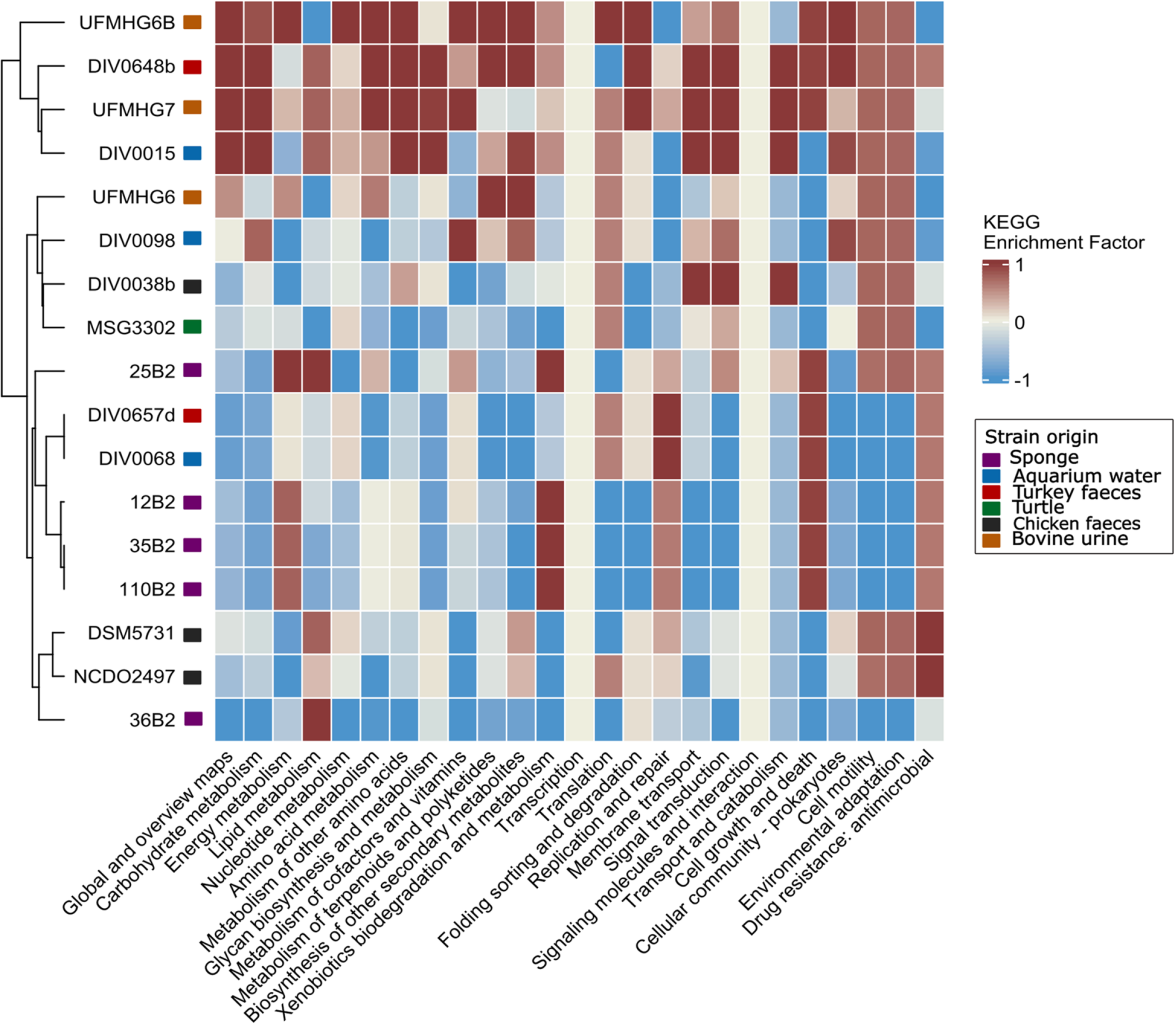
KEGG pathway enrichment heatmap of chromosomal genes in the *V. fluvialis* genomes. The genomes are ordered according to the dendrogram, constructed using hierarchical clustering based on the enrichment matrix. The coloured squares next to the strain names represent the strain origin

To better understand the differences amongst the *V. fluvialis* genomes, the intraspecies enrichment of each chromosomal sequence in the different KEGG pathway groups was determined and displayed as a heatmap in Fig. 2. There was no clear link between the clustering of the genomes according to their functional profile and their habitat. Notably, the genomes of the sponge-isolated strains 25B2 and 36B2 were functionally different from the genomes of the three other sponge-isolated strains, which were near-identical. The latter were clustered with strains DIV0657d and DIV0068, which were identical, although they were isolated from a turkey and aquarium water, respectively. Several KEGG pathway groups were generally enriched in the sponge-isolated genomes. The “energy metabolism” group includes in the sponge-isolated genomes 10 additional genes compared to the other strains, involved in oxidative phosphorylation (KO2117 to KO2124, different subunits of a V/A-type H + /Na + -transporting ATPase) and methane metabolism (KO0121, S-(hydroxymethyl) glutathione dehydrogenase / alcohol dehydrogenase, and KO1070, S-formylglutathione hydrolase). The enrichment in other KEGG pathway groups is due to a lesser number of additional genes. The “xenobiotics biodegradation and metabolism” group is enriched in 2 genes involved in styrene degradation (KO1039-KO1040, glutaconate CoA-transferase), the “replication and repair” group by a gene involved in DNA replication and mismatch repair (KO3111, single-strand DNA-binding protein), the “cell growth and death” group by a gene involved in the cell cycle (KO1358, ATP-dependent Clp protease involved in the cell cycle), and the “antimicrobial drug resistance” group by a gene involved in cationic antimicrobial peptide resistance (KO1448¸N-acetylmuramoyl-L-alanine amidase). Among the sponge-isolated V. fluvialis strains, the strain 25B2 was relatively enriched in the KEGG pathway groups “xenobiotics biodegradation and metabolism”, as it contained an additional gene coding for an alcohol dehydrogenase (K13954) involved in chloroalkane and naphthalene degradation; “signal transduction”, which included 13 additional genes coding for two-component systems involved in chemotaxis (K00575, K01546-K01548, K02405-K02406, K02556, K03406-K03408, K03412-K03413, and K14205) as well as a potassium-transporting ATPase; “cell motility”, which included 26 additional genes involved in flagellar assembly (K02387-K02392, K02396-K02397, K02400-KO2401, K02406-K02412, K02416-K02417, K02419-K02422, and K02556-K02557) and 13 genes involved in chemotaxis (K00575, K02410, K02416-K02417, K02556-K02557, K03406-K03408 and K03410-K03413); and “environmental adaptation” with an additional flagellin (K02496). The strain 36B2 was relatively enriched in the KEGG pathway groups “glycan biosynthesis and metabolism”, as it contained two additional genes involved in glycosphingolipid biosynthesis (K07407) and in glycan degradation (K12111); and “membrane transport”, as it included 3 additional genes involved in ABC transporters (K10188-K10190) and 4 involved in the phosphotransferase system (K02745-K02747 and K02768). Both strains 25B2 and 36B2 were depleted in the functions “nucleotide metabolism” relative to the three other strains, which included genes involved in the purine and pyrimidine metabolism (K01081), beta-alanine metabolism (K00128), taurine and hypotaurine metabolism (K15024), selenocompound metabolism (K00549), cyanoamino acid metabolism (K05349), and glutathione metabolism (K0033).

**Fig. 3:**
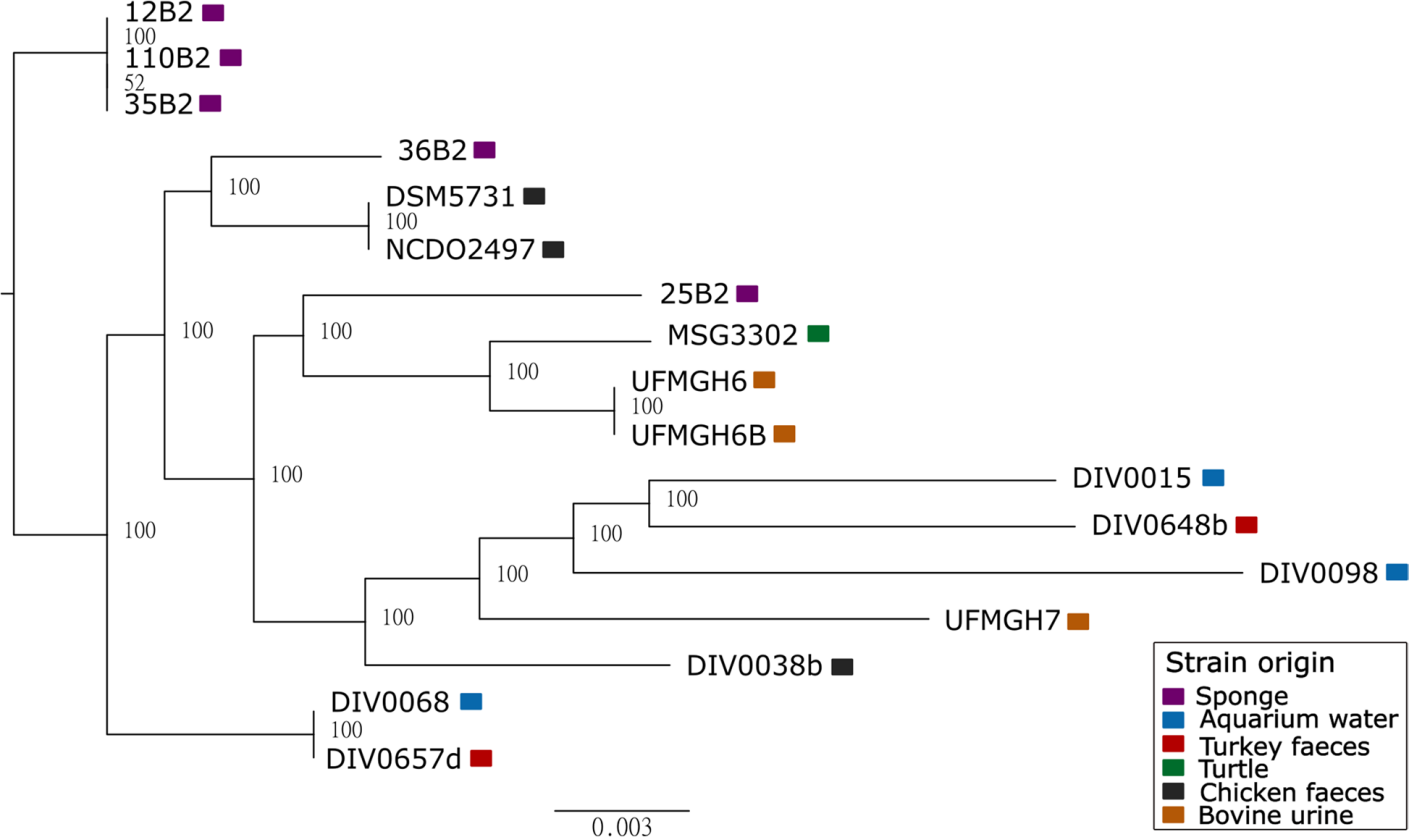
Maximum likelihood phylogenetic tree of the 1824 core genes of the *V. fluvialis* genome, with bootstrap values indicated by the nodes. The coloured squares next to the strain names represent the strain origin

A total of 6563 genes were identified in the 17 genomes (Supplementary Figure S3), of which 1824 belonged to the core genome (present in all 17), 1752 to the shell (present in 2 to 16) and 2987 were unique (present in only 1). The phylogenetic tree of the core genes concatenation does not display separate clades linked to the strain’s origin (Fig. 3). However, the unique genes do reflect their specific habitat. Seventeen genes were present in the genome of each of the five sponge-isolated strains and absent from all others: an endoribonuclease L-PSP (yabJ); a prophage transcriptional repressor (cro/c1 HTH DNA-binding domain); a putative phage holin; 7 subunits of a V-type sodium ATPase (ntpA, ntpB, ntpC, ntpD, ntpE, ntpG and ntpK); an ATP synthase subunit (atpE); a glycine betaine transporter (*opuD*); a serine endopeptidase; an L-lysine 6-monooxygenase (*cdr2*); a putative sulphate transporter; a putative antisigma factor antagonist; and 2 universal stress proteins (*nhaX*).

**Fig. 4:**
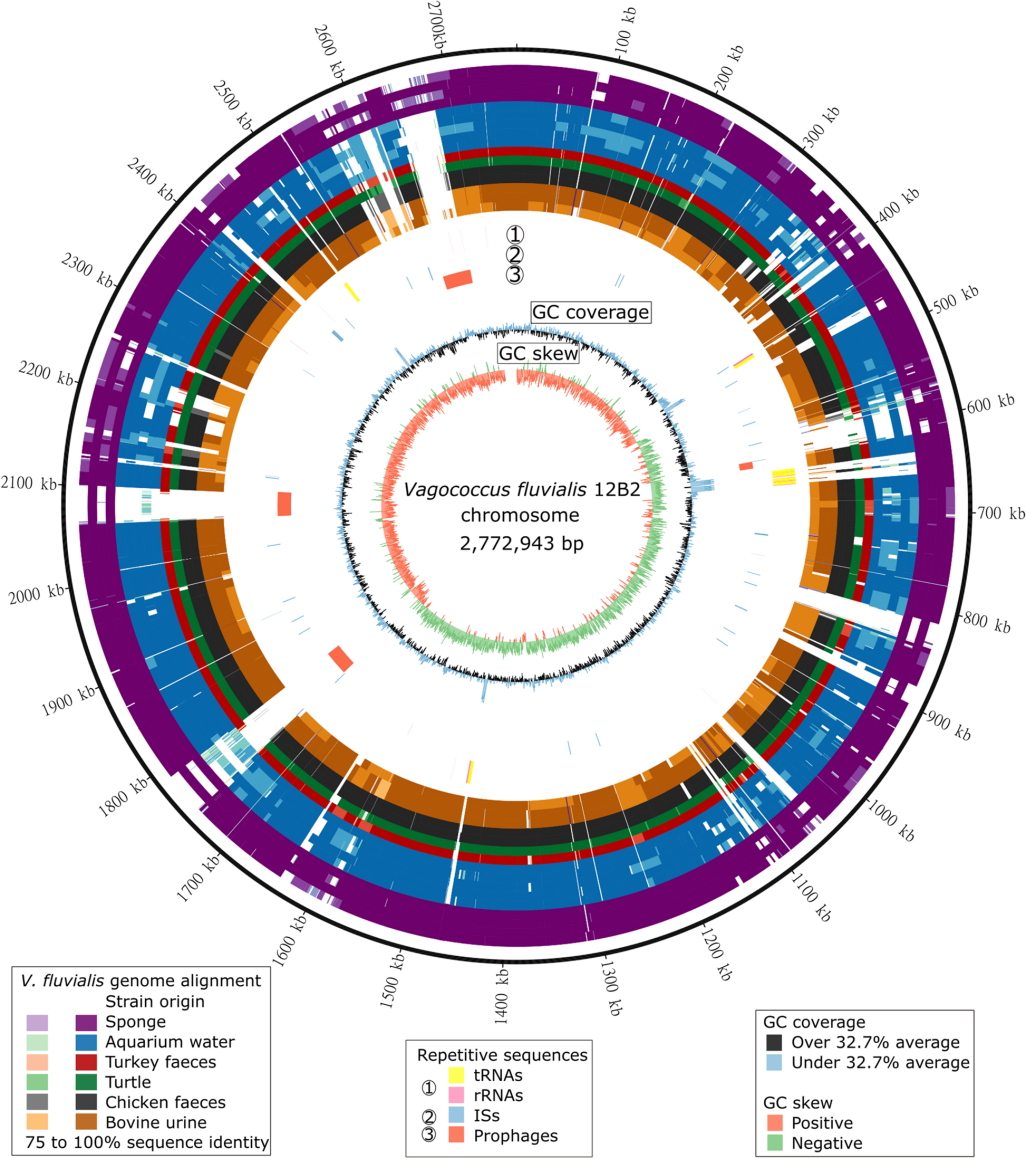
Circular genome map of *V. fluvialis* 12B2. The 16 outer rings represent the shared nucleotide identities of NUCmer whole-genome pairwise alignments with the other *V. fluvialis* genomes (from outer to inner: 25B2, 35B2, 36B2, 110B2, DIV0015, DIV0068, DIV0098, DIV0648b, DIV0657d, MSG3302, DIV0038b, DSM5731, NCDO2497, UFMGH6, UFMGH6B and UFMGH7), shown in colour scale from 75 to 100%. Rings numbered 1 to 3 represent repetitive sequences found in the genome: (1) RNA gene sequences (tRNAs and rRNAs), (2) IS sequences, and (3) prophage sequences. The inner rings represent GC content and GC skew

Alignment of the *V. fluvialis* genomes unveiled that most regions present in some sponge-isolated strains (outer 4 rings, purple) but absent from all other *V. fluvialis* genomes (inner 12 rings) were insertion sequences and prophages (Fig. 4).

### Characterization of MGEs and their abundance

**Fig. 5:**
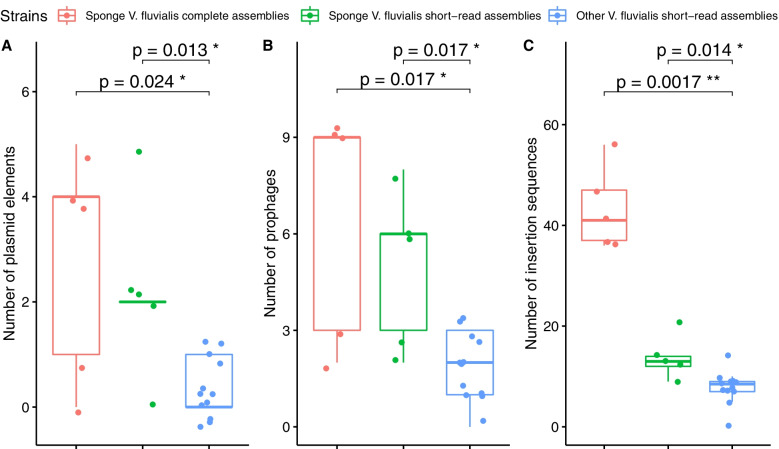
Abundance of MGEs in the *V. fluvialis* genomes. Boxplots showing the abundance of [A] plasmid elements, [B] prophages, and [C] insertion sequences (ISs) in the genomes of the 5 sponge-isolated *V. fluvialis* strains (red), the short-read assemblies of the genomes of the 5 sponge-isolated *V. fluvialis* strains (green), and the 12 other *V. fluvialis* genomes (blue). Boxes represent the interquartile range (IQR) and the horizontal line indicates the position of the median. Each dot represents one data point. The p-value of the statistical test on the means is indicated; their difference is significant when *p* < 0.05 (* = significant, ns = not significant)

All *V. fluvialis* genomes were screened for MGEs (plasmid-associated elements, prophage, and insertion sequences). Our comparative analysis revealed significant difference in abundance of MGEs between the complete genome sequences of sponge-isolated strains and the previously sequenced strains (Figs. 4 and 5). This was still the case in the short-read fragmented assembly of the genome of sponge isolates.

#### Plasmids

Multiple extrachromosomal circular contigs were assembled for the different strains (4 for 12B2, 3 for 35B2, 1 for 110B2 and 25B2, and 4 for 36B2). We screened these contigs for plasmid-related genes (Supplementary Table S3). Three near-identical 135 kb circular contigs (named with the strain name followed by c1; 12B2_c1, 35B2_c1 and 110B2_c1) did not contain any plasmid replication or transfer genes but were verified to be circular as long reads spanned the circular contig without any obvious break (Supplementary Figure S4). Omitting these circular contigs resulted in a marked decrease of the k-mer completeness of the three assemblies (Supplementary Figures S5, S6 and S7; see also Discussion). These sequences contained prophages and will be further discussed in Prophages section.

We identified respectively 3, 2, 0, 1 and 4 circular plasmid sequences in the genomes of strains 12B2, 35B2, 110B2, 25B2, and 36B2; each of these plasmids was named with the strain name followed by p then a number. Most of these plasmids contained regions similar to those of plasmids of other lactic acid bacteria (Supplementary Table S4), with the exception of 36B2_p1 (discussed further). Notably, plasmid 36B2_p4 shared 99.73% identity over 85% of its sequence with *Enterococcus faecalis* strain L12 plasmid pL12-B.

Plasmid replication and transfer genes were identified in the extrachromosomal circular contigs. Replication protein genes (*repA* and *repB*) were detected in 7 plasmids and had 75 to 87% sequence identity with their closest hit, which were all *rep* genes of *Enterococcus faecium* or *Lactococcus lactis* plasmids (Supplementary Table S5). Relaxase protein genes were detected in 4 plasmids and were homologous to a relaxase protein of *Staphylococcus aureus* plasmid pC221_p4 (Supplementary Table S6). An origin of transfer (*oriT*) was identified in only one plasmid, 36B2_p1 (Supplementary Table S7).

We identified a 48-kb conjugative plasmid, 36B2_p1 (Supplementary Figure S8) assigned to the mobility group MOBQ and type IV secretion system (T4SS) mating pair formation class MPFT. Except for one mobilizable plasmid (36B2_p2), all other plasmids identified in the genomes were annotated as non-mobilizable.

#### Prophages

We detected prophages in all but one (DIV0098) genomes of *V. fluvialis*. A total of 51 prophage sequences were finally annotated in the 17 V*. fluvialis* genomes (Supplementary Table S8). Similarities were found with phage sequences of other lactic acid bacteria. Several clusters of identical chromosomal prophage sequences were identified phylogenetically (Supplementary Figure S9). All prophages of 12B2, 35B2 and 110B2 were identical between the three strains. Additionally, identical prophages were found in the genomes of DIV0068 and DIV0657d, and in those of UFMGH6 and UFMGH6B.

As detailed in Fig. 5B, the number of prophage sequences identified in the sponge-isolated *V. fluvialis* genomes was significantly higher than in previously sequenced strains. The former accounted for 31 of the 51 prophage sequences identified in *V. fluvialis* (i.e. 60%). Prophages covered 3 to 5.6% of the sponge-isolated *V. fluvialis* genomes and 0 to 3.4% of those from other sources.

**Fig. 6:**
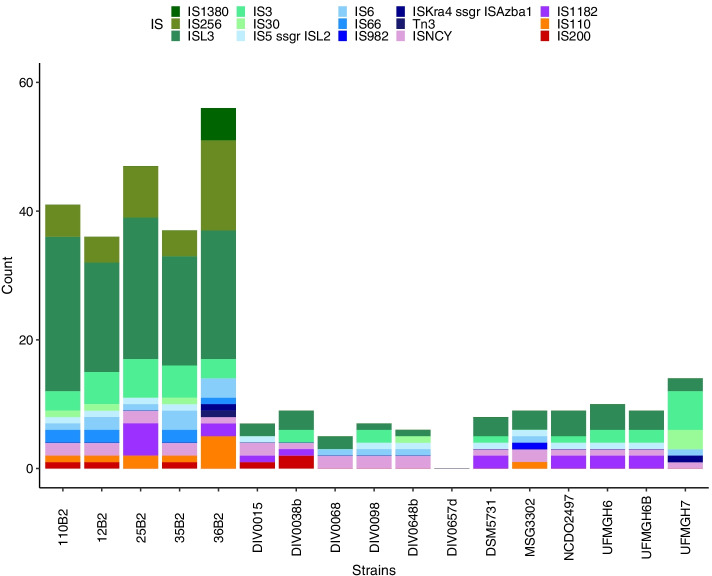
Number and type of insertion sequences in each *V. fluvialis* genome. Represented in a stacked barplot, with IS types colour coded by Tpase type: DDE, blue and green colours; undetermined, pink and purple; DEDD, orange; HUH, red

Although no replication elements were detected in the prophage-rich circular 135 kb sequence present in the genome of three sponge-isolated *V. fluvialis* strains (12B2_c1/35B2_c1/110_c1; Supplementary Figure S4; described in the previous Sect. 3.1), the GC skew shows a sharp transition at 0 and 45 kb. The predicted prophage regions covered over 75% of the sequence (Supplementary Table S8). Some of the remaining regions were predicted as phage remnants, as they were identified by only one of the phage prediction programs used (data not shown; illustrated in dark green in ring [3] of Fig. 6). Three pairs of phage attachment sites (*attL* and *attR*) were identified. Genes involved in both the lytic (holin) and lysogenic (integrase, recombinase) cycles were detected in the sequence.

#### Insertion sequences (ISs)

In total, 311 insertion sequences were detected in the 17 V*. fluvialis* genomes. These belonged to a variety of families, containing 3 of the 4 transposase types (DDE, DEDD and HUH; not serine). As shown in Fig. 5, the abundance of ISs in the sponge-isolated *V. fluvialis* genomes was significantly higher than in the other genomes of the same species. Indeed, the five genomes assembled in this study contained 70% (217 ISs; 36 in 12B2, 37 in 35B2, 41 in 110B2, 47 in 25B2 and 56 in 36B2) of all ISs detected in the *V. fluvialis* genomes. The total count of insertion sequences per genome as well as the distribution amongst IS families, and subgroups, when possible, is shown in Fig. 6. Insertion sequences were found in all genomes, ranging from five to 56 total sequences, except for strain DIV0657d in which no IS sequence was detected.

The IS family ISL3 accounted for most elements annotated (41%, 128 ISs), with 0 to 9 different elements (data not shown). A Tn3 IS was found uniquely in the genome of strain 36B2. The IS families IS256, IS66 and IS110 were detected exclusively in sponge-isolated strains genomes.

## Discussion

Previous analyses of sponge-associated bacteria were based on bins obtained from metagenomic binning and often restricted to analyses of gene content [15, 16]. Although one previous study revealed an enrichment in mobile genetic elements in sponge obligate symbionts, no systematic mobilome comparison between closely related sponge-associated and free-living strains of a given species was conducted till now.

Our study revealed a unique functional profile of *Vagococcus fluvialis* genomes within the Enterococcaceae family (Fig. 1). The functional grouping of the strains matched phylogeny rather than habitat (Figs. 1 and 3). For example, marine *V. fluvialis* strains were functionally closer to strains of the same species found in turkey or cows than to other marine *Vagococcus* spp. strains. This echoes a previous report that did not detect any functional differences in the genomes of sponge-associated strains of three bacterial genera compared to congeneric free-living strains [17]. The most enriched pathways in *V. fluvialis* were directly related to defence mechanisms (antimicrobial drug resistance and xenobiotics degradation; Supplementary Figure S2). Other relatively abundant pathways, ABC transporters (membrane transport) and two-component signal transduction systems, may have coevolved as resistance determinants against antimicrobial peptides in Firmicutes [18]. *V. fluvialis* intraspecies divergence was also not related to the isolation source of the different strains. For example, although the species was described as motile [6], four sponge-isolated, the turkey-isolated and the aquarium-isolated strains were depleted in cell motility genes (Fig. 2). More specifically, these strains lack the bacterial chemotaxis genes present in the other *V. fluvialis *genomes. The loss of chemotaxis and motility genes in bacteria associated to marine sponges has previously been described and is hypothesized to be caused, and indication of, a mutualistic lifestyle and vertical transmission of symbionts [19, 20]. Pangenome analysis of *V. fluvialis *revealed great intraspecies diversity (Supplementary Figure S3): notably, the *V. fluvialis* genomes shared only 28% of core genes, whereas 45.5% of orthologous genes were unique. The unique genes found in sponge-isolated strains coded for a variety of proteins considered to be linked to a marine lifestyle, such as a glycine betaine transporter, previously reported to be linked to osmoadaptation [21], and a serine protease, which participates to salt tolerance [22]. Over a third of the unique genes found in the sponge-isolated strains code for the different subunits of a V-type sodium ATPase. These genes are also greatly responsible for the enrichment in energy metabolism of the sponge-isolated strains compared to those of other habitats (Fig. 2). The V-type sodium-transporting ATPase has been characterized in marine bacteria, which therefore have the ability to pump sodium ions outward [23–26]. Other functions found to be enriched in sponge-isolated strains have also previously been reported in studies of marine sponge bacteria, such as formaldehyde detoxification [27] and styrene degradation [28, 29]. Although the mentioned functions differentiate the sponge-isolated strains from those of other sources, there are also differences among the sponge-isolated strains in functions such as chemotaxis, flagellar assembly, and ABC transporters. Therefore, no clear functional grouping could be observed related to strain source (Fig. 2).

Our analysis showed a significantly higher abundance of MGEs in the sponge-isolated *V. fluvialis* genomes compared to those of previously sequenced strains (Fig. 5). These results are consistent with other studies reporting numerous active mobile genetic elements (MGEs) in sponge-associated bacteria, involved in ecological fitness and adaptation [4, 30, 31]. More generally, these large numbers of MGEs are characteristic of genomes of symbiotic bacteria [32]. The enrichment of the replication and repair KEGG pathway in the sponge-isolated genomes compared to the others (Fig. 2) is an indication of this high abundance of MGEs.

This is the first report describing plasmids, including one conjugative plasmid, in the species *Vagococcus fluvialis*. The only previously sequenced *Vagococcus* plasmids were a 15-kb plasmid in the genome of *V. teuberi* strain DSM21459 [33] and two 62 and 45-kb plasmids in the genome of *V. carniphilus* strain ATCC BAA-640. However, our analysis of previously published *V. fluvialis* genomes as well as of representative genomes of other *Vagococcus* species showed that plasmid replication elements are also present in some of these genomes (Fig. 5). High-quality complete assembly of these genomes will probably lead to the identification of additional plasmid sequences. Moreover, the influence of these plasmids on the phenotype of the *Vagococcus fluvialis* strains should be further investigated to gain insight into their function.

Although a total of ten plasmids were identified in the different sponge-isolated *V. fluvialis* genomes, only 2 were predicted to be mobilizable. By comparison, about half of the plasmid sequences in GenBank are non-mobilizable [34]. More specifically, in the case of Firmicutes, 65% of analysed plasmids were non-mobilizable, 30% mobilizable and 5% conjugative. The conjugative plasmid 36B2_p1 carried a plasmid secretion system (MPFT) typical of plasmids of Proteobacteria, which are known to transfer to Firmicutes [34]. No similarity was found between this plasmid and sequences in the NCBI database (Supplementary Table S4), suggesting the novelty of this element. Among the non-conjugative plasmids, plasmid 36B2_p2 was classified as a mobilizable and assigned to the mobility group MOBP (Supplementary Table S3), which is the most represented mobility group in plasmid databases [34]. The non-conjugative plasmids assembled in this study are aligned in size and GC with those found in Firmicutes [35]. Conjugative plasmids in lactic acid bacteria are usually larger (> 30 kb) than mobilizable and non-mobilizable plasmids, in accordance with our findings (Supplementary Table S3) [11].

Phages are a critical part of bacterial genomes that can provide beneficial functions for bacteria such as adaptation to new environmental niches and acquisition of antibiotic resistance [36, 37]. According to a large-scale study, prophages, i.e. chromosome integrated lysogenic phages, are present in 46% of bacterial genomes [38]. In this study, prophage sequences in *V. fluvialis* genomes were analysed using five different methods (cited in Methods “Annotation” section), which yielded very variable results due to the difficulty of annotating these regions [39]. Prophage regions detected by one method but not others (and therefore not considered as prophages) are probably phage remnants [38, 40]. We detected prophages in all but one genomes of *V. fluvialis*. Although these were mostly related to phages found in other lactic acid bacteria, the percentage identity was low (under 50%, Supplementary Table S8), indicating sequence novelty. On average, prophage sequences make up 3.1% of a typical genome [38, 40, 41]. In sponge-isolated *V. fluvialis* genomes, this percentage ranged between 3 to 5.6%. The presence of CRISPRs in the genomes (Table 1) is a testament to the viral infections sustained by sponge-associated bacteria [2]. A 135 kb circular extrachromosomal sequence, composed of numerous prophage elements (Supplementary Figure S4, Supplementary Table S8), was found in three sponge-isolated *V. fluvialis* genomes. We hypothesise that this sequence (12B2_c1/35B2_c1/110B2_c1) is derived from an excised circular phage DNA. Such circular prophage sequences have been previously reported [42, 43]: notably, an extrachromosomal contig corresponding to an excised chromosome-encoded prophage was detected in the complete genome sequence of *Lactobacillus hokkaidonensis* [44].

Insertion sequences (ISs) are widespread in prokaryotic genomes and are involved in genome plasticity and gene inactivation [45, 46]. A large-scale analysis of ISs in bacterial genomes showed that Firmicutes harbour a median of 12 ISs per genome (ranging from 0 to 91) [45]. This value matches the number of ISs found in the previously sequenced *V. fluvialis* genomes, whereas sponge-isolated strains contain up to five times that amount (Fig. 6). Certain IS families (IS256, IS66 and IS110) were found exclusively in the latter. IS256 has been linked to biofilm phenotypic variability in *S. aureus* [47]. It is also abundant in *Enterococcus faecalis* and *E. faecium* and linked to their antimicrobial resistance [48]. In the *V. fluvialis* genomes, certain ISs were also found in regions flanking antimicrobial resistance genes (data not shown). IS66 contains 3 ORFs, including a transposase and 2 genes possibly modulating its translation [46]. The presence of these insertion sequences may be linked to the adaptation of the bacterial strains to the sponge host as their transposition can result in gene inactivation and modulation of surrounding gene expression, respectively. Such genetic variability may promote adaptation to environmental changes [49].

## Conclusions

In summary, the complete assemblies of *V. fluvialis* strains obtained in this study allowed the correct quantification and identification of repetitive sequences. The significantly larger abundance of MGEs is the most notable distinction between sponge-isolated strains and those isolated from other sources. This could be linked to the microbial density and complexity found in marine sponges [1]. Further investigation into MGEs found in the sponge microbiome, but also in the sponge cells, could give more insight into the interactions and genetic exchange taking place.

## Methods

### Bacterial strains

In this study, we analysed five *Vagococcus fluvialis* strains previously isolated from the marine sponges *Halichondria panicea* (strains 35B2 and 36B2) and *Hymeniacidon perlevis* (strains 12B2, 25B2 and 110B2) [14]*.* Strains were grown overnight in BHI Broth (Merck, Darmstadt, Germany) at 37 °C with agitation (180 rpm). Genomic DNA was extracted using the Gentra Puregene Yeast/Bacteria Kit (Qiagen, Hilden, Germany) following the DNA purification protocol for gram-positive bacteria as specified in the manufacturer’s instructions. DNA purity was assessed using a Nanodrop Spectrophotometer (Thermo Fisher Scientific, USA) and dsDNA was quantified using a Qubit (Thermo Fisher Scientific, USA).

### Genome sequencing

Long-read sequencing libraries were constructed using the Ligation Sequencing Kit SQK-LSK109 and the Native Barcoding Expansion 1–12 EXP-NBD104 (Oxford Nanopore Technologies, Oxford, United Kingdom) according to the manufacturer’s instructions. The library was subsequently loaded onto a primed R9.4.1 SpotON Flow Cell on a MinION device (Oxford Nanopore Technologies, Oxford, United Kingdom). Sequencing was run for 36 h using the MinKNOW software 19.06.8 for data acquisition and Guppy v4 for basecalling. Adapter trimming and barcode demultiplexing (75% barcode sequence identity threshold) of generated reads was performed using Porechop v0.2.4 (https://github.com/rrwick/Porechop). Short-read data was using Illumina NovaSeq 6000 sequencing (paired-end, 2 × 100 bp) at the Brussels Interuniversity Genomics High Throughput core (http://www.brightcore.be/), Belgium. The quality of short and long reads was assessed using FastQC v0.11.9 (https://www.bioinformatics.babraham.ac.uk/projects/fastqc/).

### Genome assembly and quality assessment

The genomes of all sponge-associated strains were assembled in a hybrid fashion using Unicycler v0.4.8 [50], except for strain 110B2, for which the completeness of the assembly obtained using Unicycler was below par compared with the other strains. To solve this, the long read data were first assembled with Flye v2.8.2 [51], followed by Unicycler polishing using the short NovaSeq reads. We assessed the quality of the assemblies using several methods. Short and long reads were mapped to the genome assemblies using minimap2 and the output was subsequently visualized in Tablet v1.17.08.17 [52, 53]. Coverage evenness and hard-clips were manually inspected. The contiguity of assembly graphs was visually inspected using Bandage [54]. K-mer frequency distributions and assembly completeness were assessed using K-mer Analysis Toolkit (KAT) v2.4.2 [55]. Genome completeness was assessed using BUSCO v5.0.0 with the Firmicutes odb10 database [56] and CheckM with the c_Bacilli marker lineage [57]. Whole genome taxonomic annotation was performed using GTDB-Tk [58].

For their comparison with short-read-based published genomes, these five genomes were also assembled using exclusively the Illumina NovaSeq paired-end short reads with SPAdes v3.15.2 [59]. This assembly yielded 126, 173, 120, 165 and 236 contigs for the genomes of 12B2, 25B2, 35B2, 36B2 and 110B2, respectively.

### Annotation

The genomes were automatically annotated using the Prokka annotation pipeline v1.14.6 [60]. The 16S rRNA gene sequences in the genomes were extracted using Barrnap v0.9 (https://github.com/tseemann/barrnap). Additional functional annotation was achieved using eggNOG-mapper [61, 62]. CRISPR-Cas systems were detected by the CRISPRCasFinder web server [63]. Insertion sequences were detected using the ISsaga web application pipeline [64, 65]. Prophages were predicted using the following programs and web servers: PHASTER [66], Prophinder [67], PhiSpy [68], ProphageHunter [69], and Phigaro [70]. Only prophages identified by at least 2 of the 5 methods were retained. Plasmid-related genes were detected using several methods: *rep* genes (PlasmidFinder 2.1; Gram Positive database, threshold settings of minimum 50% identity and 20% coverage [71]) and *oriT* and relaxase genes (oriTfinder; threshold settings of minimum 0.1 e-value for BLAST and 0.001 for HMMer [72]). MOB-typer was used to characterize the mobility of the predicted plasmids [73]. Plasmids were identified as “conjugative” when both a mobility type (MOB) and a mating pair formation machinery (MPF) could be predicted, as “mobilizable” when a MOB group could be predicted but not an MPF, and as “non-mobilizable” when neither could be predicted.

### Functional profiling of the Enterococcaceae family

All *Vagococcus* spp. genomes and all reference genomes of the Enterococcaceae family available on September 18^th^ 2021 were retrieved from the NCBI Genome database for functional comparison (Supplementary Table S9). Proteins from these and from the newly sequenced *V. fluvialis* genomes were submitted to GhostKOALA for KEGG Orthology (KO) functional annotation using the “genus_prokaryotes” KEGG database [74–77]. The KO abundance matrix was standardized relative to genomes using the Hellinger method [78]. A principal component analysis (PCA) was performed on the correlation matrix of the standardized abundance matrix using the vegan package (https://CRAN.R-project.org/package=vegan) in R v4.0.5.

An enrichment analysis of the main bacterial KEGG pathway groups was performed. The number of genes related to each pathway group for each genome was standardized by average and standard deviation of each group. This resulted in an “Enrichment Factor” ranging from -1 to 1, representing respectively the under- or over-representation of genes related to KEGG pathway groups in a given genome compared to the other genomes. Heatmaps were created with Euclidean clustering using the ComplexHeatmap package v2.6.2. in R v4.0.5 [79].

### Comparative genome analysis

The genomes of thirteen *Vagococcus fluvialis* strains and eleven representative genomes of other *Vagococcus* species (Supplementary Table S1) were used for comparison with the newly sequenced genomes. A whole-genome Average Amino Acid Identity (AAI) matrix of all *V. fluvialis* genomes, as well as the representative genomes of other *Vagococcus* species, was calculated (http://enve-omics.ce.gatech.edu/g-matrix/index). The genome of *V. fluvialis* bH819 had only 73% AAI with all other *V. fluvialis* genomes (Supplementary Figure S1). KoT species delimitation using a core gene alignment confirmed that this genome does not belong to the *V. fluvialis* species (Supplementary Figure S10) [80], and we therefore excluded it from subsequent intraspecific comparisons. NUCmer pairwise whole-genome alignments were performed for each *V. fluvialis* genomes using the 12B2 genome as reference [81]. Circular genome plots were produced using Circos v0.69–8 [82] and Inkscape v1.1 (https://inkscape.org/).

We carried out a pan-core genome study for *Vagococcus fluvialis* using Roary 3.13.0. All genomes were subjected to identical annotation protocols to generate comparable data sets for each genome. All-against-all bidirectional BLASTP alignments with a sequence identity higher than 95% were inputted in the Roary pipeline to build an ortholog table [83]. The number of orthologous gene clusters for core, shell and unique genome profiles was extracted from the Roary output. The same method was used to carry out a pan-core genome study for *V. fluvialis* using the short-read assemblies of the five newly sequenced genomes instead of the complete hybrid assemblies (Supplementary Figure S11).

Heatmaps were created using the ComplexHeatmap package v2.6.2. and barplots using the ggplot2 package v3.3.3 in R v4.0.5 [79].

### Phylogenetic trees

Core genes and chromosomal prophage sequences were aligned using MAFFT v7.475 [84]. The core genes and chromosomal prophage sequence alignments contained 1,736,517 and 110,742 nucleotide sites, with 95.15 and 30.68% being constant sites (containing a single character state over all sequences), respectively. Unrooted maximum-likelihood phylogenetic trees were computed with 1000 bootstrap repetitions using IQ-TREE v2.1.2 [85, 86]. ModelFinder Plus was used to find the best parameters [87] by computing the log-likelihoods of an initial parsimony tree for many different models and choosing the model that minimizes the Bayesian information criterion (BIC) score. The model chosen was GTR + F + R3 for the core gene tree, and GTR + F + I + G4 for the prophage sequence tree. Trees were visualised with midpoint rooting using Figtree v1.4.4 (https://github.com/rambaut/figtree/tree/v1.4.4).

### MGE abundance comparison

We compared the abundance of mobile genetic elements (MGEs) in the five presently constructed genomes (“sponge_fluvialis”: 12B2, 25B2, 35B2, 36B2 and 110B2), the short-read assemblies of the same genomes (“sponge_fluvialis_spades”), and the previously sequenced *V. fluvialis* genomes (“other_fluvialis”: DSM5731, NCDO2497, UFMGH6, UFMGH6B, UFMGH7, DIV0015, DIV0038b, DIV0068, DIV0098, DIV0648b, DIV0657d and MSG3302). The medians and interquartile ranges (IQR) were represented in boxplots. The normality of the data was assessed using the Shapiro–Wilk’s test. Statistical difference between abundance means was computed with the unpaired two-sample Wilcoxon test with a significance level α = 0.05. This analysis was carried out using the dplyr (https://CRAN.R-project.org/package=dplyr) and ggpubr packages (https://CRAN.R-project.org/package=ggpubr) in R v.4.0.5.

## Supplementary Information

**Additional file 1: Supplementary Table S1.** Characteristics of the thirteen *Vagococcus fluvialis* genomes available online and of the representative genomes of other *Vagococcus* genera. **Supplementary Figure S1.** Average amino acid identity matrix of the *V. fluvialis* genomes and the representative genomes of other *Vagococcus* species. **Supplementary Figure S2.** KEGG functional category enrichment heatmap of chromosomal genes in Enterococcaceae genomes. The genomes are ordered according to the dendrogram, constructed using hierarchical clustering based on the enrichment matrix. **Supplementary Figure S3.** Gene presence/absence matrix of all orthologous gene clusters in the *V. fluvialis* pan genome. The genomes are ordered according to the dendrogram constructed using hierarchical clustering based on the gene presence/absence matrix. Genes shown in blue represent the core genome (present in all *V. fluvialis* genomes), those in green represent the shell genomes (present in more than 2 genomes), and those in yellow are unique genes (present in only one genome). **Supplementary Table S3.** Characteristics of all extrachromosomal circular sequences identified in the genomes of the five sponge-isolated *V. fluvialis* strains. Sequence length, Rep genes (number), relaxase gene presence, origin of transfer (oriT) presence, mobility, MOB group and MPF type. **Supplementary Figure S4.** Prophage-encoding circular sequence c1 map. The 3 outer rings represent prophage related elements. From outer to inner (1) phage attachment sites, (2) phage function annotation, and (3) predicted prophage regions. The inner rings (4) and (5) represent GC content and GC skew. **Supplementary Figure S5.** KAT plot of the genome assembly of strain 12B2. Left: assembly without extrachromosomal contig 12B2_c1. Right, assembly including 12B2_c1. **Supplementary Figure S6.** KAT plot of the genome assembly of strain 35B2. Left: assembly without extrachromosomal contig 35B2_c1. Right, assembly including 35B2_c1. **Supplementary Figure S7.** KAT plot of the genome assembly of strain 110B2. Left: assembly without extrachromosomal contig 110B2_c1. Right, assembly including 110B2_c1. **Supplementary Table S4.** Best blast hits of all extrachromosomal circular sequences. Sequences not reported in the table did not have any hit (12B2_c1, 35B2_c1, 36B2_p1, and 110B2_c1). **Supplementary Table S5.** Rep genes encoded in the plasmids: Rep group, position, closest hit, plasmid host of closest Rep protein hit and identity percentage. Results retrieved from PlasmidFinder. **Supplementary Table S6.** Relaxase protein genes encoded in the plasmids: protein length, gene coordinates and homolog BLASTp identity percentage. **Supplementary Table S7.** Origin of transfer and relaxase protein of the plasmid 36B2_p1: oriT coordinates, size and sequence and relaxase protein length, gene coordinates and BLASTp identity percentage. **Supplementary Figure S8.** Circular map of the conjugative plasmid 36B2_p1. From outer to inner ring, these represent (1) conjugation-related genes, (2 and 3) COG functional categories, (4) GC content and (5) GC skew. **Supplementary Table S8**. Prophage annotation results. Results for each genome sequence of prophage detection using Phaster, ProphageHunter, Phigaro, PhiSpy and Prophinder. **Supplementary Figure S9.** Maximum likelihood phylogenetic tree of all chromosomal prophage sequences identified in all *V. fluvialis* genomes, with bootstrap support values indicated by the nodes. **Supplementary Table S9.** Detailed list of the reference genomes of the Enterococcaceae family and all *Vagococcus* spp. genomes, used for functional comparison. **Supplementary Figure S10**. KoT species delimitation of the V. fluvialis genomes and the representative genomes of other *Vagococcus *species. **Supplementary Figure S11.** Gene presence/absence matrix of all orthologous gene clusters in the V. fluvialis pan-genome, using the short-read assemblies of the sponge-isolated *V. fluvialis* strains genomes (sr). The genomes are ordered according to the dendrogram constructed using hierarchical clustering based on the gene presence/absence matrix. Genes shown in blue represent the core genome (present in all *V. fluvialis* genomes), those in green represent the shell genomes (present in more than 2 genomes), and those in yellow are unique genes (present in only one genome).

## Data Availability

The raw Oxford Nanopore and Illumina NovaSeq reads were deposited in NCBI SRA under the BioProject accession PRJNA755170. The genome assemblies of newly sequenced strains were deposited in GenBank under the accession numbers: CP081472-CP081476 (*Vagococcus fluvialis* 12B2), CP081470-CP081471 (*Vagococcus fluvialis* 25B2), CP081466-CP081468 (*Vagococcus fluvialis* 35B2), CP081461-CP081365 (*Vagococcus fluvialis* 36B2), and CP081459-CP081460 (*Vagococcus fluvialis* 110B2). All other genome sequences analysed during the current study are available from the National Centre for Biotechnology Information (NCBI) genome database. The multiple sequence alignment and phylogenetic trees in Newick format used to create the phylogenetic trees in this study were shared on Figshare: https://doi.org/10.6084/m9.figshare.19949804.
