# Supplementary material for: Comparative genome analysis of Vagococcus fluvialis reveals abundance of mobile genetic elements in sponge-isolated strains
Source: BMC Genomics. 2022 Aug 25;23:618. doi: 10.1186/s12864-022-08842-9 (PMC9413892; doi:10.1186/s12864-022-08842-9)
Supplement: Supplementary file 1 — Additional file 1: Supplementary Table S1. Characteristics of the thirteen Vagococcus fluvialis genomes available online and of the representative genomes of other Vagococcus genera. Supplementary Figure S1. Average amino acid identity matrix of the V. fluvialis genomes and the representative genomes of other Vagococcus species. Supplementary Figure S2. KEGG functional category enrichment heatmap of chromosomal genes in Enterococcaceae genomes. The genomes are ordered according to the dendrogram, constructed using hierarchical clustering based on the enrichment matrix. Supplementary Figure S3. Gene presence/absence matrix of all orthologous gene clusters in the V. fluvialis pan genome. The genomes are ordered according to the dendrogram constructed using hierarchical clustering based on the gene presence/absence matrix. Genes shown in blue represent the core genome (present in all V. fluvialis genomes), those in green represent the shell genomes (present in more than 2 genomes), and those in yellow are unique genes (present in only one genome). Supplementary Table S3. Characteristics of all extrachromosomal circular sequences identified in the genomes of the five sponge-isolated V. fluvialis strains. Sequence length, Rep genes (number), relaxase gene presence, origin of transfer (oriT) presence, mobility, MOB group and MPF type. Supplementary Figure S4. Prophage-encoding circular sequence c1 map. The 3 outer rings represent prophage related elements. From outer to inner (1) phage attachment sites, (2) phage function annotation, and (3) predicted prophage regions. The inner rings (4) and (5) represent GC content and GC skew. Supplementary Figure S5. KAT plot of the genome assembly of strain 12B2. Left: assembly without extrachromosomal contig 12B2_c1. Right, assembly including 12B2_c1. Supplementary Figure S6. KAT plot of the genome assembly of strain 35B2. Left: assembly without extrachromosomal contig 35B2_c1. Right, assembly including 35B2_c1. Supplementary Figure [file 12864_2022_8842_MOESM1_ESM.pdf]

Supplementary Table S1. Characteristics of the thirteen *Vagococcus fluvialis* genomes available online and of the representative genomes of other *Vagococcus* genera.

Strain	Accession number	Origin	Sequencing technology	Assembly	Coverage	Scaffolds	Size (Mb)
NCDO2497	NGJX01	Chicken faeces	Illumina Miseq	SPAdes v 3.10.1	197.1x	28	2.65
DSM5731	QPJV01		Illumina HiSeq	SPAdes v 3.10.1	553x	29	2.65
bH819	FWFD01	Cheese	Not specified	Not specified	379x	22	2.95
UFMG-H6	JAAVMC01	Bovine urinary samples	Illumina NextSeq	SPAdes v 3.11.1	182x	40	2.68
UFMG-H6B	JAAVMV01				38x	44	2.85
UFMG-H7	JAAVMB01				165x	41	2.99
DIV0015	JAFLWJ01	Aquarium water (zoo)	Illumina Hiseq 2500 2X250	CLC genomics workbench v. v8.0.3	73x	21	2.88
DIV0648b	JAFLWN01	Turkey faeces			129x	27	3.13
MSG3302	JAFLWP01	Turtle cloacal swab			159x	40	2.76
DIV0098	JAFLWM01	Aquarium water (zoo)			59x	32	2.68
DIV0038b	JAFLWK01	Chicken faeces			56x	53	2.83
DIV0657d	JAFLWO01	Turkey faeces			53x	69	2.74
DIV0068	JAFLWL01	Aquarium water (zoo)			124x	72	2.74
Vagococcus teuberi DSM 21459	CP017267 CP017268	Malian artisanal sour milk fene	PacBio	CLC NGS Cell v8.0	400	2 (1chr, 1p)	2.18 (plasmid 15-Kb)
Vagococcus silagei 2B-2	SDGV01	Silage brewer's grain	Illumina MiSeq	CLC NGS Cell v10	455	51	2.53
Vagococcus salmoninarum NCFB 2777	NGJU01	Diseased adult rainbow trout (Oncorhynchus mykiss)	Illumina MiSeq	SPAdes v3.10.1	245.9	45	3.10
Vagococcus penaei LMG 24833	NGJV01	Spoilage microbiota of cooked shrimp (Penaeus vannamei)	Illumina MiSeq	SPAdes v3.10.1	306.2	52	2.34
Vagococcus martis D7T301_1_1	MVAB01	Marten small intestine (Martes flavigula)	PacBio	RS HGAP v3.0	475	3	2.56

Vagococcus lutrae LBD1	AYSH01	Gastrointestinal tract of a Largemouth Bass (Micropterus salmoides)	Illumina	CLC Genomics Worbench v4.9	720	20	1.83
Vagococcus humatus JCM 31581	PXZH01	Soil beneath a decomposing pig carcass	Illumina MiSeq	SPAdes v3.11.1	120.3	10	2.1
Vagococcus fessus CCUG 41755	NGJY01	Dead seal liver and kidney (Phoca vitulina)	Illumina MiSeq	SPAdes v3.10.1	306	8	2.26
Vagococcus entomophilus DSM 24756	NGJZ01	Wasp digestive tract (Vespula vulgaris)	Illumina MiSeq	SPAdes v3.10.1	299.4	7	2.44
Vagococcus elongatus CCUG 51432	NGKA01	Swine-manure storage pit	Illumina MiSeq	SPAdes v3.10.1	259.6	55	2.88
Vagococcus carniphilus ATCC BAA-640	CP060720 CP060721 CP060722	Ground beef	PacBio	HGPA v3.0	330	3 (1chr, 2p)	3.02 (plasmids 62 & 45-kb)
Vagococcus acidifermentans LMG 24798	NGKC01	Acidogenic fermentation bioreactor	Illumina MiSeq	SPAdes v3.10.1	272.5	40	2.86

	V.silagei.2B-2	V.penae.LMG24833	V.fluvialis.bH819	V.carniphilus.ATCCBAA-640	V.teuberi.DSM21459	V.martis.D7T301	V.salmoninarum.NCFB2777	V.fessus.CCUG41755	V.humatus.JCM31581	V.entomophilus.DSM24756	V.lutrae.LBD1	V.elongatus.CCUG51432	V.acidifermentans.LMG24798	V.fluvialis.DIV0038b	V.fluvialis.25B2	V.fluvialis.NCDO2497	V.fluvialis.DSM5731	V.fluvialis.36B2	V.fluvialis.UFMGH7	V.fluvialis.UFMGH6B	V.fluvialis.UFMGH6	V.fluvialis.MSG3302	V.fluvialis.DIV0657d	V.fluvialis.DIV0068	V.fluvialis.35B2	V.fluvialis.12B2	V.fluvialis.110B2	V.fluvialis.DIV0098	V.fluvialis.DIV0015	V.fluvialis.DIV0648b
V.silagei.2B-2	100	66	63	64	63	63	57	57	58	56	58	56	59	64	64	63	63	64	63	63	63	64	64	64	63	63	63	64	63	63
V.penae.LMG24833	66	100	65	65	66	65	60	58	59	58	59	57	61	66	66	66	66	66	65	66	66	66	66	66	66	66	66	66	66	65
V.fluvialis.bH819	63	65	100	76	67	66	59	59	59	57	59	57	61	73	73	73	73	73	72	73	73	73	73	73	73	73	73	73	72	72
V.carniphilus.ATCCBAA-640	64	65	76	100	67	66	59	59	59	57	59	57	61	74	74	74	74	74	74	74	74	74	74	74	74	74	74	74	74	74
V.teuberi.DSM21459	63	66	67	67	100	96	59	59	59	59	59	58	62	67	66	67	67	66	67	67	67	67	67	67	67	67	67	67	67	66
V.martis.D7T301	63	65	66	66	96	100	59	58	60	58	59	58	62	66	66	66	66	65	65	66	66	66	66	66	66	66	66	66	66	66
V.salmoninarum.NCFB2777	57	60	59	59	59	59	100	65	62	60	63	60	59	60	60	60	60	60	59	59	60	60	60	60	59	59	59	60	60	59
V.fessus.CCUG41755	57	58	59	59	59	58	65	100	61	59	62	59	58	58	59	59	59	58	59	59	59	59	59	58	59	59	59	59	58	58
V.humatus.JCM31581	58	59	59	59	59	60	62	61	100	59	61	60	59	59	59	59	59	59	59	59	59	59	59	59	59	59	59	59	59	59
V.entomophilus.DSM24756	56	58	57	57	59	58	60	59	59	100	58	57	59	58	58	58	58	58	58	58	58	58	58	58	57	57	57	58	58	57
V.lutrae.LBD1	58	59	59	59	59	59	63	62	61	58	100	62	59	60	59	59	59	59	59	59	59	60	59	59	59	59	59	59	60	59
V.elongatus.CCUG51432	56	57	57	57	58	58	60	59	60	57	62	100	62	57	57	58	58	58	57	57	58	58	57	57	57	57	57	58	57	57
V.acidifermentans.LMG24798	59	61	61	61	62	62	59	58	59	59	59	62	100	61	61	61	61	61	61	61	61	61	61	61	61	61	61	61	61	60
V.fluvialis.DIV0038b	64	66	73	74	67	66	60	58	59	58	60	57	61	100	98	98	98	98	97	98	98	98	98	98	98	98	98	97	98	97
V.fluvialis.25B2	64	66	73	74	66	66	60	59	59	58	59	57	61	98	100	98	98	98	97	98	98	98	98	98	98	98	98	97	97	97
V.fluvialis.NCDO2497	63	66	73	74	67	66	60	59	59	58	59	58	61	98	98	100	100	99	98	98	98	98	98	98	98	98	98	97	98	97
V.fluvialis.DSM5731	63	66	73	74	67	66	60	59	59	58	59	58	61	98	98	100	100	99	98	98	98	98	98	98	98	98	98	97	98	97
V.fluvialis.36B2	64	66	73	74	66	65	60	58	59	58	59	58	61	98	98	99	99	100	97	98	98	98	98	98	98	98	98	97	97	97
V.fluvialis.UFMGH7	63	65	72	74	67	65	59	59	59	58	59	57	61	97	97	98	98	97	100	97	97	97	97	97	97	97	97	97	97	97
V.fluvialis.UFMGH6B	63	66	73	74	67	66	59	59	59	58	59	57	61	98	98	98	98	98	97	100	100	99	98	98	98	98	98	97	97	97
V.fluvialis.UFMGH6	63	66	73	74	67	66	60	59	59	58	59	58	61	98	98	98	98	98	97	100	100	99	98	98	98	98	98	97	97	97
V.fluvialis.MSG3302	64	66	73	74	67	66	60	59	59	58	60	58	61	98	98	98	98	98	97	99	99	100	97	97	98	98	98	97	97	97
V.fluvialis.DIV0657d	64	66	73	74	67	66	60	59	59	58	59	57	61	98	98	98	98	98	97	98	98	97	100	100	98	98	98	97	97	97
V.fluvialis.DIV0068	64	66	73	74	67	66	60	58	59	58	59	57	61	98	98	98	98	98	97	98	98	97	100	100	98	98	98	97	97	97
V.fluvialis.35B2	63	66	73	74	67	66	59	59	59	57	59	57	61	98	98	98	98	98	97	98	98	98	98	98	100	100	100	97	97	97
V.fluvialis.12B2	63	66	73	74	67	66	59	59	59	57	59	57	61	98	98	98	98	98	97	98	98	98	98	98	100	100	100	97	97	97
V.fluvialis.110B2	63	66	73	74	67	66	59	59	59	57	59	57	61	98	98	98	98	98	97	98	98	98	98	98	100	100	100	97	97	97
V.fluvialis.DIV0098	64	66	73	74	67	66	60	59	59	58	59	58	61	97	97	97	97	97	97	97	97	97	97	97	97	97	97	100	98	97
V.fluvialis.DIV0015	63	66	72	74	67	66	60	58	59	58	60	57	61	98	97	98	98	97	97	97	97	97	97	97	97	97	97	98	100	98
V.fluvialis.DIV0648b	63	65	72	74	66	66	59	58	59	57	59	57	60	97	97	97	97	97	97	97	97	97	97	97	97	97	97	97	98	100

Supplementary Figure S1. Average amino acid identity matrix of the *V. fluvialis* genomes and the representative genomes of other *Vagococcus* species.

Supplementary Table S2. General chromosomal features of the short-read assemblies of the genomes of sponge-isolated *V. fluvialis* strains.

Genome	Contigs	Chromosome length (Mb)	CDSs	tRNAs	rRNAs (5S, 16S, 23S)
12B2	65	2.92	2999	34	3 (1,1,1)
35B2	59	2.92	2997	33	3 (1,1,1)
110B2	182	2.93	2984	34	3 (1,1,1)
25B2	86	2.72	2704	35	3 (1,1,1)
36B2	89	2.82	2791	38	3 (1,1,1)

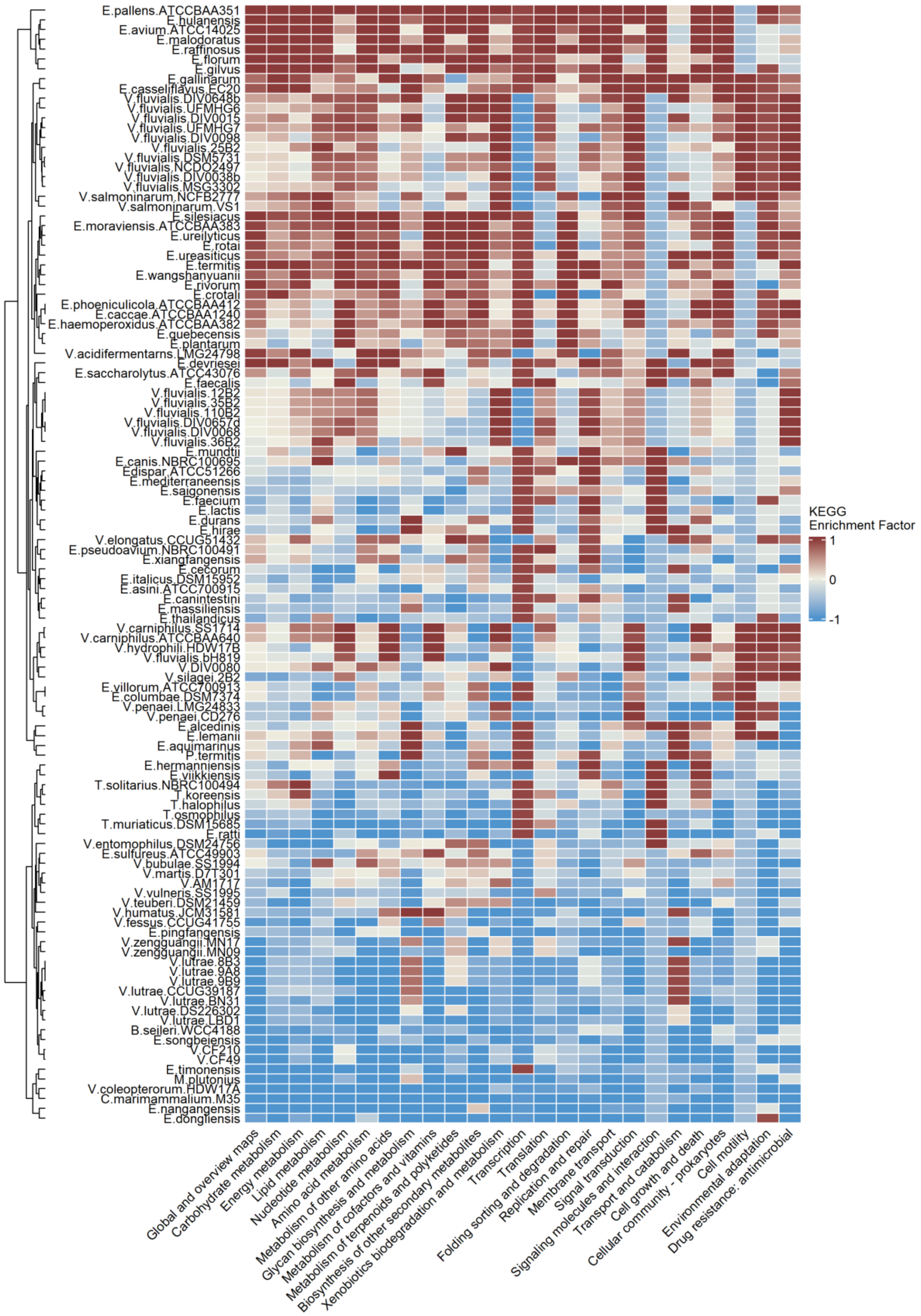

Supplementary Figure S2. KEGG functional category enrichment heatmap of chromosomal genes in Enterococcaceae genomes. The genomes are ordered according to the dendrogram, constructed using hierarchical clustering based on the enrichment matrix.

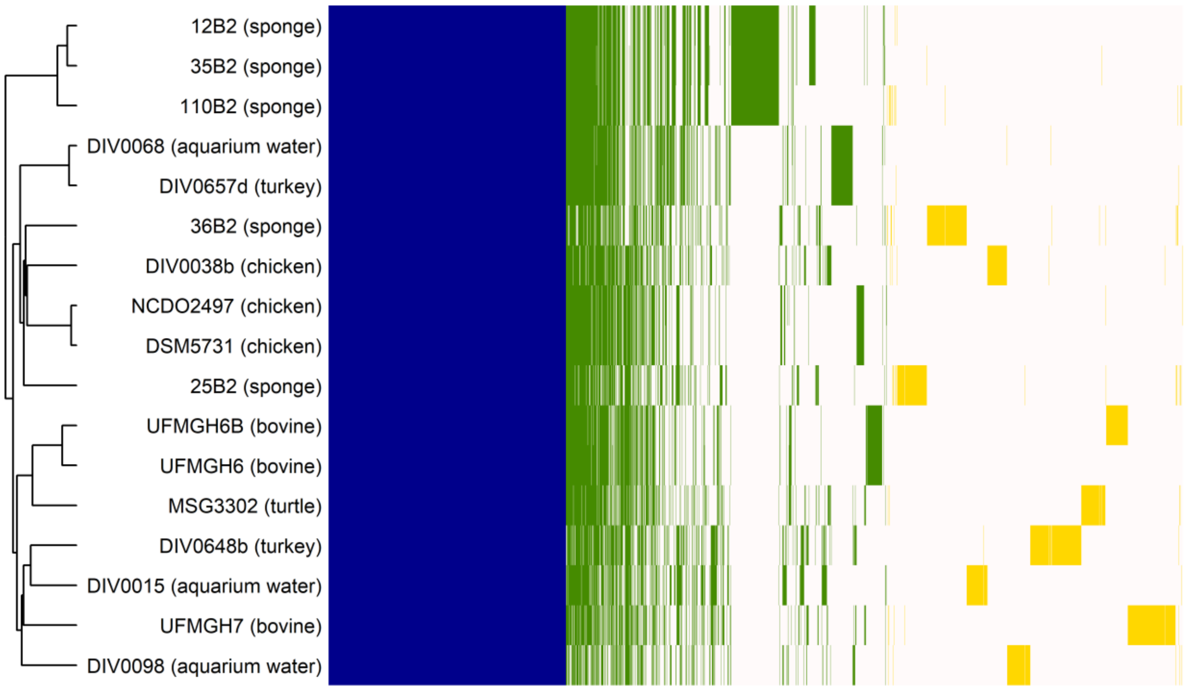

Supplementary Figure S3. Gene presence/absence matrix of all orthologous gene clusters in the *V. fluvialis* pan-genome. The genomes are ordered according to the dendrogram constructed using hierarchical clustering based on the gene presence/absence matrix. Genes shown in blue represent the core genome (present in all *V. fluvialis* genomes), those in green represent the shell genomes (present in more than 2 genomes), and those in yellow are unique genes (present in only one genome).

Supplementary Table S3. Characteristics of all extrachromosomal circular sequences identified in the genomes of the five sponge-isolated *V. fluvialis* strains. Sequence length, Rep genes (number), relaxase gene presence, origin of transfer (*oriT*) presence, mobility, MOB group and MPF type.

Sequence	Length (kb)	Rep (no.)	Relaxase	*oriT*	Mobilizable	MOB	MPF
12B2_c1	135.6				No		
12B2_p1	31.2				No		
12B2_p2	10.5	*repA*	Yes		No		
12B2_p3	6.7	*repA* (2)			No		
35B2_c1	135.2				No		
35B2_p1	12.9	*repA*			No		
35B2_p2	10.5	*repA* (2)	Yes		No		
110B2_c1	135.7				No		
25B2_p1	11.1		Yes		No		
36B2_p1	48.6		Yes	Yes	Conjugative	MOBQ	MPFT
36B2_p2	12.3	*rep*			Yes	MOBP	
36B2_p3	11.4	*repB*			No		
36B2_p4	4.5	*repA*			No		

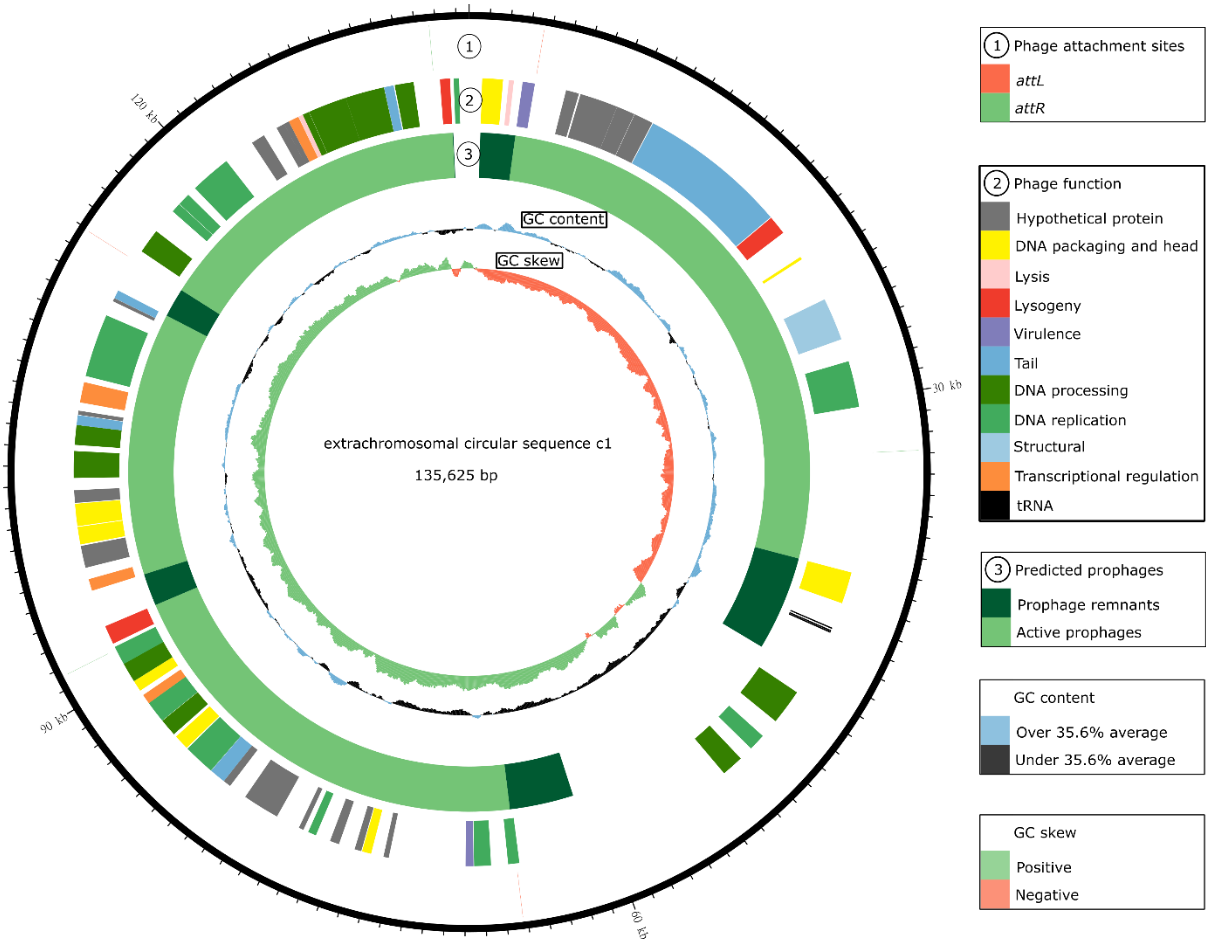

Supplementary Figure S4. Prophage-encoding circular sequence c1 map. The 3 outer rings represent prophage-related elements. From outer to inner (1) phage attachment sites, (2) phage function annotation, and (3) predicted prophage regions. The inner rings (4) and (5) represent GC content and GC skew.

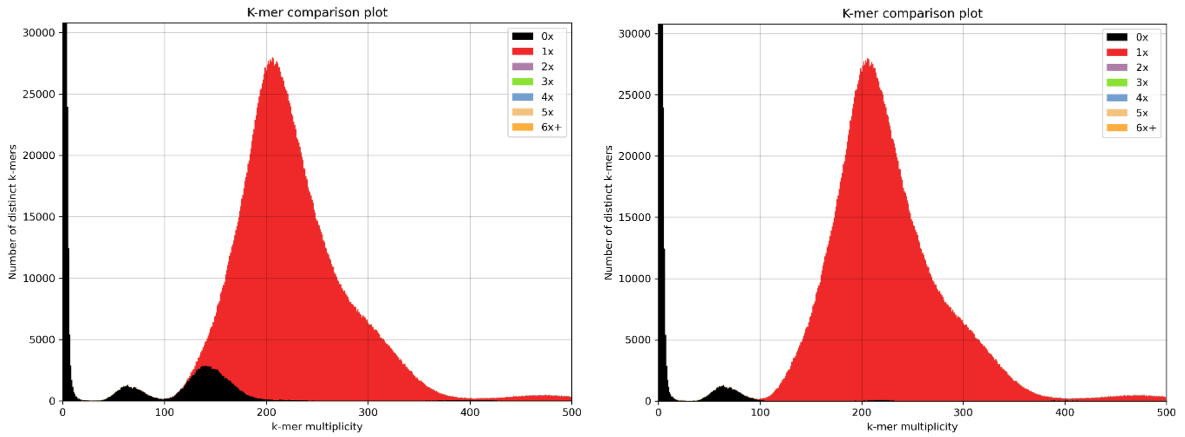

Supplementary Figure S5. KAT plot of the genome assembly of strain 12B2. Left: assembly without extrachromosomal contig 12B2_c1. Right, assembly including 12B2_c1.

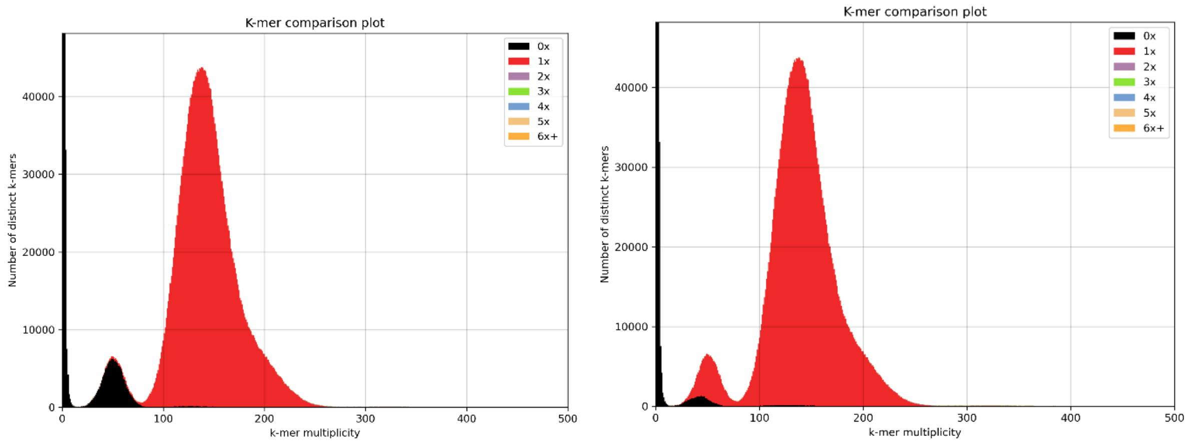

Supplementary Figure S6. KAT plot of the genome assembly of strain 35B2. Left: assembly without extrachromosomal contig 35B2_c1. Right, assembly including 35B2_c1.

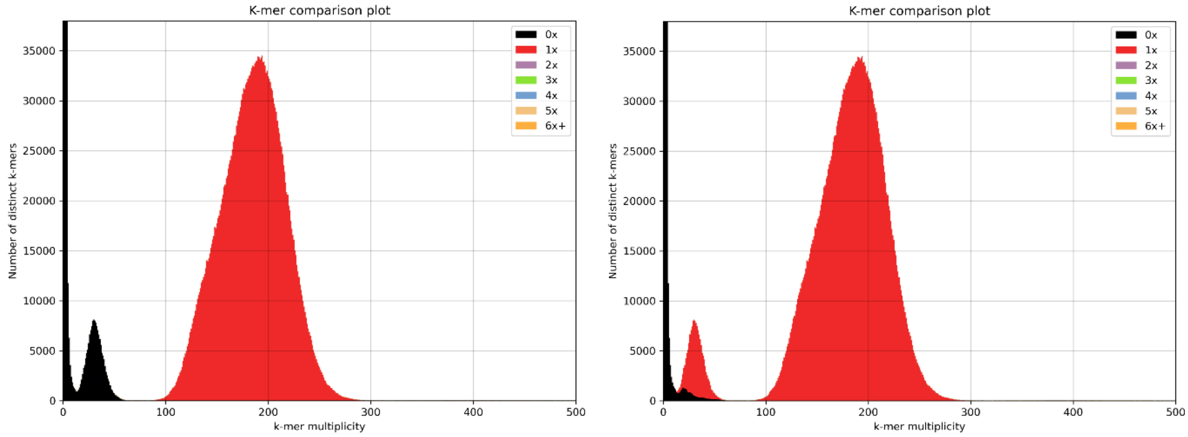

Supplementary Figure S7. KAT plot of the genome assembly of strain 110B2. Left: assembly without extrachromosomal contig 110B2_c1. Right, assembly including 110B2_c1.

Supplementary Table S4. Best blast hits of all extrachromosomal circular sequences. Sequences not reported in the table did not have any hit (12B2_c1, 35B2_c1, 36B2_p1, and 110B2_c1).

Plasmid	Best hit	Query Cover	E. value	Per. Ident	Acc. Len	Accession
12B2_p1	*Enterococcus faecium* strain VB3378 plasmid pVB3378_p2, complete sequence	2%	2e-49	68.48%	44920	CP072709.1
12B2_p2	*Enterococcus faecalis* strain L12 plasmid pL12-B, complete sequence	26%	0.0	99.59%	4717	CP072512.1
12B2_p3	*Enterococcus* sp. strain BCCO 40_GJ1 plasmid pGJTET1, complete sequence	16%	0.0	84.22%	6973	KY887560.1
35B2_p1	*Enterococcus faecalis* strain L12 plasmid pL12-B, complete sequence	9%	0.0	99.34%	4717	CP072512.1
35B2_p2	*Enterococcus faecalis* strain L12 plasmid pL12-B, complete sequence	26%	0.0	99.59%	4717	CP072512.1
25B2_p1	*Vagococcus hydrophili* strain HDW17B chromosome, complete genome	20%	0.0	74.22%	3045783	CP049887.1
36B2_p2	*Enterococcus durans* strain FDAARGOS 1437 plasmid unnamed3, complete sequence	48%	0.0	89.33%	11726	CP077323.1
36B2_p3	*Enterococcus faecalis* isolate 28157_4#260 genome assembly, plasmid: 2	46%	0.0	99.92%	80750	LR962441.1
36B2_p4	*Enterococcus faecalis* strain L12 plasmid pL12-B, complete sequence	85%	0.0	99.73%	4717	CP072512.1

Supplementary Table S5. Rep genes encoded in the plasmids: Rep group, position, closest hit, plasmid host of closest Rep protein hit and identity percentage. Results retrieved from PlasmidFinder.

Plasmid	Rep group	position	Rep protein	Origin	Identity
12B2_p2	rep11a	3198..3576	repA(pB82)	*Enterococcus faecium*	76.76
12B_p3	rep11a	1230..1610	repA(pB82)	*Enterococcus faecium*	76.77
	rep18b	6652..7178	repA(pEF418)	*Enterococcus faecium*	75.94
35B2_1	rep11a	3156..3535	repA(pB82)	*Enterococcus faecium*	76.3
35B2_2	rep11a	3570..3950	repA(pB82)	*Enterococcus faecium*	76.76
	rep18b	8495..9021	repA(pEF418)	*Enterococcus faecium*	75.94
36B2_2	rep33	9418..10522	rep(pK214)	*Lactococcus lactis*	75.22
36B2_3	repUS41	9051..10275	repB(PML21)	*Enterococcus faecalis*	87.4
36B2_4	rep11a	1076..1747	repA(pB82)	*Enterococcus faecium*	75.22

Supplementary Table S6. Relaxase protein genes encoded in the plasmids: protein length, gene coordinates and homolog BLASTp identity percentage.

Plasmid	Protein length	Gene coordinate	Homolog	BLASTp id
12B2_p2	295 aa	8547..9434 (+)	*Staphylococcus aureus* pC221_p4	41.97%
35B2_p2	295 aa	06239..7126 (-)	*Staphylococcus aureus* pC221_p4	41.97%
25B2_p1	452 aa	6872..8230 (-)	*Staphylococcus aureus* pC221_p4	41.13%

Supplementary Table S7. Origin of transfer and relaxase protein of the plasmid 36B2_p1: *oriT* coordinates, size and sequence and relaxase protein length, gene coordinates and BLASTp identity percentage.

oriT			Relaxase		
coordinate	size	sequence	Protein length	Gene coordinate	BLASTp id
44680..44726 (-)	47 bp	gtcatagaataattctttgttttcttcaatcgattcaatagaaatat	654 aa	42207..44171 (-)	99.39%

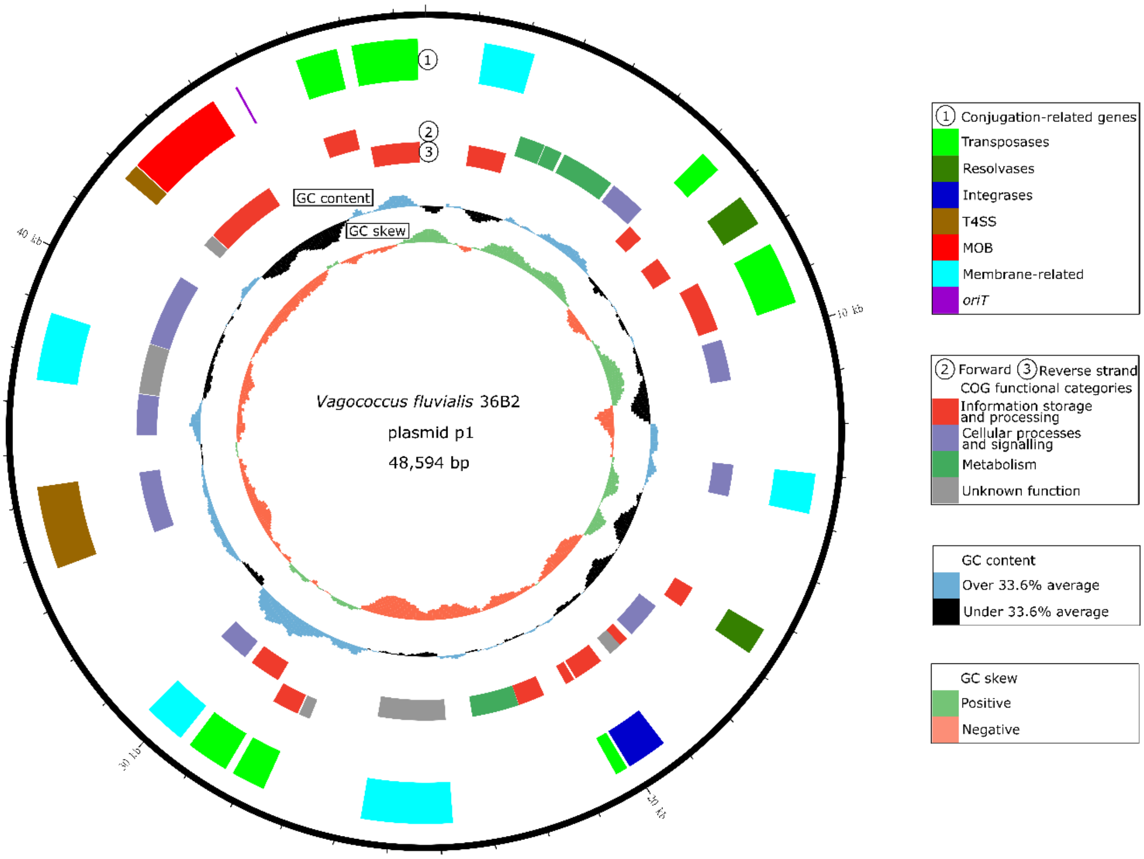

Supplementary Figure S8. Circular map of the conjugative plasmid 36B2_p1. From outer to inner ring, these represent (1) conjugation-related genes, (2 and 3) COG functional categories, (4) GC content and (5) GC skew.

Supplementary Table S8. Prophage annotation results. Results for each genome sequence of prophage detection using Phaster, ProphageHunter, Phigaro, PhiSpy and Prophinder.

Genome	Phage	Start	End	PHASTER	tRNA	Total protein	Phage protein	Hypothetical protein	attsite	Best phage hits	Identity %	GC %	ProphageHunter	Closest phage	Gene number	Phigaro	Taxonomy	PhiSpy	Prophinder
12B2	1	611864	624587	YES	0	8	6	2	YES	PHAGE_Staphy_SPbeta_like_NC_029119	50	32.77	YES	Streptococcus phage phiJH1301-2	20	NO		NO	NO
	2	1742691	1787822	YES	0	73	40	33	NO	PHAGE_Entero_phiFL2A_NC_013643, PHAGE_Bacill_vB_BtS_BMBtp14_NC_048640	5.47	34.11	YES	Lactococcus phage 56701	49	YES	Siphoviridae	NO	YES
	3	2060897	2105283	YES	0	58	36	22	YES	PHAGE_Lister_B025_NC_009812	15.51	33.46	YES	Staphylococcus phage ROSA	62	YES	Siphoviridae	NO	YES
	4	2634370	2686339	YES	1	59	41	18	YES	PHAGE_Strept_315.2_NC_004585	16.94	33.16	YES	Streptococcus phage JX01	60	YES	Siphoviridae	YES	YES
12B2_c1	1	2934	32845	YES	0	13	8	5	YES	PHAGE_Geobac_E3_NC_029073	30.76	37.08	YES	Lactobacillus phage Ld3	11	NO		NO	NO
	2	21609	39463	NO									YES	Aeromonas phage 65.2	21	YES	Myoviridae	NO	NO
	3	65220	93043	YES	0	34	18	16	YES	PHAGE_Lister_P100_NC_007610, PHAGE_Entero_EFP01_NC_047796, PHAGE_Entero_EFDG1_NC_029009, PHAGE_Brevib_Sundance_NC_028749, PHAGE_Lister_A511_NC_009811, PHAGE_Lister_LP_048_NC_024359, PHAGE_Lister_vB_LmoM_AG20_NC_020871, PHAGE_Entero_EfV12_phi1_NC_048087, PHAGE_Geobac_E3_NC_029073	5.88	34.71	YES	Bacillus phage Basilisk	27	NO		NO	NO
	4	95118	112059	YES	0	22	13	9	NO	PHAGE_Clostr_c_st_NC_007581	31.81	36.30	YES	Enterococcus phage EFP01	73	NO		NO	YES

	5	113922	134576	YES	0	17	11	6	YES	PHAGE_Lactoc_WRP3_NC_027341, PHAGE_Lactoc_phiL47_NC_023574, PHAGE_Entero_phiEF24C_NC_009904	17.64	35.68	YES	Lactococcus phage WRP3	38	NO		NO	NO
35B2	1	414429	459560	YES	0	73	40	33	NO	PHAGE_Bacill_vB_BtS_BMBtp14_NC_048640, PHAGE_Entero_phiFL2A_NC_013643	5.47	34.11	YES	Lactococcus phage 56701	49	YES	Siphoviridae	NO	YES
	2	732635	777021	YES	0	58	36	22	YES	PHAGE_Lister_B025_NC_009812	15.51	33.46	YES	Staphylococcus phage ROSA	62	YES	Siphoviridae	NO	YES
	3	1306277	1358246	YES	1	59	41	18	YES	PHAGE_Strept_315.2_NC_004585	16.94	33.16	YES	Streptococcus phage JX01	60	YES	Siphoviridae	YES	YES
	4	2056714	2069437	YES	0	8	6	2	YES	PHAGE_Staphy_SPbeta_like_NC_029119	50	32.77	YES	Streptococcus phage phiJH1301-2	20	NO		NO	NO
35B2_c1	1	3919	24573	YES	0	17	11	6	YES	PHAGE_Entero_phiEF24C_NC_009904, PHAGE_Lactoc_WRP3_NC_027341, PHAGE_Lactoc_phiL47_NC_023574	17.64	35.68	YES	Lactococcus phage WRP3	52	NO		NO	NO
	2	22245	47211	YES	0	13	8	5	YES	PHAGE_Geobac_E3_NC_029073	30.76	36.82	YES	Liberibacter phage SC1	22	NO		NO	NO
	3	47099	71183	NO									YES	Aeromonas phage 65.2	21	YES	Myoviridae	NO	NO
	4	90471	118294	YES	0	34	18	16	YES	PHAGE_Lister_A511_NC_009811, PHAGE_Geobac_E3_NC_029073, PHAGE_Entero_EFP01_NC_047796, PHAGE_Entero_EfV12_phi1_NC_048087, PHAGE_Lister_LP_048_NC_024359, PHAGE_Lister_vB_LmoM_AG20_NC_020871, PHAGE_Lister_P100_NC_007610, PHAGE_Entero_EFDG1_NC_029009, PHAGE_Brevib_Sundance_NC_028749	5.88	34.71	YES	Bacillus phage Basilisk	27	NO		NO	NO

	5	120369	131944	YES	0	16	10	6	NO	PHAGE_Clostr_c_st_NC_007581	37.5	36.52	YES	Enterococcus phage EFP01	29	YES	Unknown	NO	NO
110B2	1	169654	182377	YES	0	8	6	2	YES	PHAGE_Staphy_SPbeta_like_NC_029119	50	32.77	YES	Streptococcus phage phiJH1301-2	20	NO		NO	NO
	2	1300461	1345592	YES	0	73	40	33	NO	PHAGE_Bacill_vB_BtS_BMBtp14_NC_048640, PHAGE_Entero_phiFL2A_NC_013643	5.47	34.11	YES	Lactococcus phage 56701	49	YES	Siphoviridae	NO	YES
	3	1618663	1663049	YES	0	58	36	22	YES	PHAGE_Lister_B025_NC_009812	15.51	33.46	YES	Staphylococcus phage ROSA	62	YES	Siphoviridae	NO	YES
	4	2192302	2244271	YES	1	59	41	18	YES	PHAGE_Strept_315.2_NC_004585	16.94	33.16	YES	Streptococcus phage JX01	60	YES	Siphoviridae	YES	YES
110B2_c1	1	454	29689	YES	0	9	6	3	YES	PHAGE_Geobac_E3_NC_029073	33.33	36.93	YES	Liberibacter phage SC1	22	NO		NO	NO
	2	18321	42468	NO									YES	Aeromonas phage 65.2	21	YES	Myoviridae	NO	NO
	3	62127	89950	YES	0	34	18	16	YES	PHAGE_Lister_LP_048_NC_024359, PHAGE_Lister_P100_NC_007610, PHAGE_Geobac_E3_NC_029073, PHAGE_Brevib_Sundance_NC_028749, PHAGE_Entero_EfV12_phi1_NC_048087, PHAGE_Entero_EFP01_NC_047796, PHAGE_Lister_A511_NC_009811, PHAGE_Lister_vB_LmoM_AG20_NC_020871, PHAGE_Entero_EFDG1_NC_029009(2)	5.88	34.71	YES	Enterococcus phage EFP01	71	NO		NO	NO
	4	92025	108966	YES	0	22	13	9	NO	PHAGE_Clostr_c_st_NC_007581	31.81	36.30	YES	Enterococcus phage EFP01	73	YES	Unknown	NO	YES
	5	110829	131482	YES	0	18	22	6	YES	PHAGE_Lactoc_phiL47_NC_023574, PHAGE_Lactoc_WRP3_NC_027341	22.22	35.68	YES	Lactococcus phage WRP3	45	NO		NO	NO
25B2	1	1800867	1836718	YES	0	53	43	10	YES	PHAGE_Lactob_PLE2_NC_031036	20.75	34.00	YES	Listeria phage B025	53	YES	Siphoviridae	YES	YES

	2	2536399	2582393	YES	1	58	41	17	YES	PHAGE_Bacill_BCJA1c_NC_006557	18.96	33.19	NO			YES	Siphoviridae	YES	YES
36B2	1	967545	1017913	YES	1	70	47	23	YES	PHAGE_Brocho_BL3_NC_015254, PHAGE_Entero_phiFL3A_NC_013648	22.85	33.43	NO	Enterococcus phage vB_EfaS_IME197	73	YES	Siphoviridae	NO	YES
	2	1021921	1072384	YES	1	71	41	30	YES	PHAGE_Strept_315.2_NC_004585	12.67	33.73	NO	Streptococcus phage JX01	67	YES	Siphoviridae	NO	NO
DSM5731	1	339761	361435	YES	1	33	22	11	YES	PHAGE_Brocho_BL3_NC_015254, PHAGE_Entero_phiFL2A_NC_013643, PHAGE_Entero_phiFL3A_NC_013648, PHAGE_Entero_phiFL1A_NC_013646, PHAGE_Entero_vB_IME197_NC_028671	9.09	32.08	NO			YES	Siphoviridae	NO	NO
NCDO2497	1	476	22193	YES	1	31	21	10	YES	PHAGE_Entero_phiFL3A_NC_013648, PHAGE_Entero_phiFL1A_NC_013646, PHAGE_Entero_phiFL2A_NC_013643, PHAGE_Brocho_BL3_NC_015254	9.67	32.13	NO			YES	Siphoviridae	NO	YES
UFMGH6	1	15336	44118	YES	1	23	15	8	YES	PHAGE_Brocho_BL3_NC_015254, PHAGE_Lactoc_WRP3_NC_027341, PHAGE_Entero_ECP3_NC_027335, PHAGE_Lactoc_phiL47_NC_023574, PHAGE_Entero_phiEF24C_NC_009904, PHAGE_Bacill_G_NC_023719	8.69	31.71	YES	Listeria phage LP-030-2	31	NO		NO	NO
UFMGH6B	1	15336	44118	YES	1	23	15	8	YES	PHAGE_Entero_phiEF24C_NC_009904, PHAGE_Entero_ECP3_NC_027335, PHAGE_Brocho_BL3_NC_015254, PHAGE_Bacill_G_NC_023719, PHAGE_Lactoc_WRP3_NC_027341, PHAGE_Lactoc_phiL47_NC_023574	8.69	31.71	YES	Listeria phage LP-030-2	31	NO		NO	NO

UFMGH7	1	245521	271608	YES	0	27	12	15	YES	PHAGE_Lactob_Lv_1_NC_011801	3.7	31.51	NO			NO		YES	NO
	2	14956	50073	NO									YES	Listeria phage vB_LmoS_293	70	YES	Siphoviridae	YES	NO
	3	51323	63146	YES	0	19	9	10	YES	PHAGE_Brevib_Jimmer2_NC_041976, PHAGE_Strept_M102AD_NC_028984, PHAGE_Strept_M102_NC_012884, PHAGE_Strept_APCM01_NC_029030, PHAGE_Brocho_BL3_NC_015254	10.52	34.11	YES	Enterococcus phage phiEF24C-P2	38	NO		NO	NO
DIV0015	1	217859	255924	YES	0	57	40	17	NO	PHAGE_Lactoc_98201_NC_031064, PHAGE_Lactoc_28201_NC_031013	15.78	34.34	YES	Enterococcus phage phiFL3B	87	YES	Siphoviridae	YES	NO
DIV0038b	1	18521	47228	YES	0	34	26	8	YES	PHAGE_Brocho_BL3_NC_015254	38.23	33.28	YES	Enterococcus phage vB_EfaS_IME197	50	YES	Siphoviridae	YES	NO
DIV0068	1	46134	110769	YES	1	61	42	19	YES	PHAGE_Lactoc_50101_NC_031040	22.95	31.83	YES	Lactococcus phage 63301	79	YES	Siphoviridae	YES	NO
	2	25224	33074	YES	0	63	38	25	NO	PHAGE_Entero_phiFL1A_NC_013646, PHAGE_Lister_A118_NC_003216, PHAGE_Entero_phiFL3A_NC_013648, PHAGE_Bacill_vB_BtS_BMBtp14_NC_048640	6.34	33.83	YES	Lactococcus phage 56701	61	YES	Siphoviridae	NO	NO
DIV0098	0																		
DIV0648b	1	56326	100121	YES	1	64	43	21	YES	PHAGE_Lister_LP_101_NC_024387	10.93	33.09	YES	Streptococcus phage phi3396	72	YES	Siphoviridae	NO	YES
	2	451422	458594	NO									YES	Bacillus phage Juglone	17	NO		YES	NO

	3	31902	42002	NO									YES	Lactococcus phage 56701	43	YES	Myoviridae	NO	NO
DIV0657d	1	15766	69288	YES	1	63	42	21	YES	PHAGE_Lactoc_50101_NC_031040	20.63	31.95	YES	Lactococcus phage 63301	78	YES	Siphoviridae	YES	NO
	2	1	18829	YES	0	24	14	10	NO	PHAGE_Bacill_vB_BtS_BMBtp14_NC_048640	16.66	34.58	YES	Enterococcus phage IME-EFm5	15	NO		NO	NO
	3	19032	41472	YES	0	38	24	14	NO	PHAGE_Entero_phiFL1A_NC_013646, PHAGE_Lister_A118_NC_003216, PHAGE_Entero_phiFL3A_NC_013648	10.52	33.26	YES	Lactococcus phage 56701	61	YES	Siphoviridae	NO	NO
MSG3302	1	201775	230004	YES	0	30	16	14	YES	PHAGE_Entero_phiEf11_NC_013696, PHAGE_Entero_vB_IME197_NC_028671	16.66	31.70	NO			NO		NO	YES
	2	93863	106359	NO									YES	Enterococcus phage phiFL3B	21	NO		YES	YES
	3	85545	120929	NO									YES	Clostridium phage vB_CpeS-CP51	12	NO		YES	NO

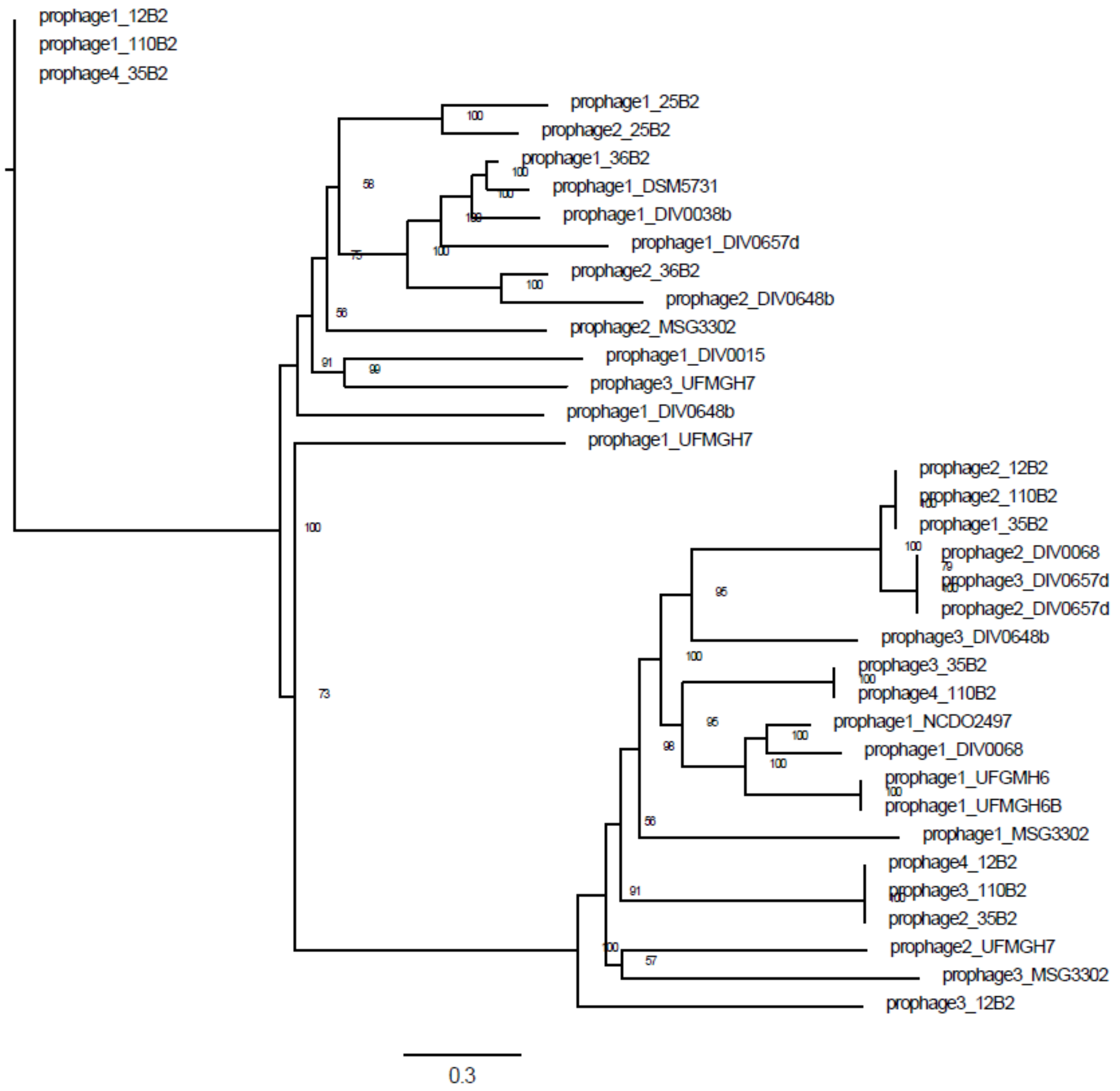

Supplementary Figure S9. Maximum likelihood phylogenetic tree of all chromosomal prophage sequences identified in all *V. fluvialis* genomes, with bootstrap support values indicated by the nodes.

Supplementary Table S9. Detailed list of the reference genomes of the Enterococcaceae family and all *Vagococcus* spp. genomes, used for functional comparison.

Organism Scientific Name	Organism Qualifier	Assembly Accession	Level	Contig N50	Size	Submission Date	BioProject	BioSample
Enterococcus casseliflavus	EC20	GCF_000157355.2	Complete Genome	3427276	3427276	11/04/2013	PRJNA32935	SAMN02596921
Catellicoccus marimammalium	M35/04/3	GCF_000313915.1	Contig	153794	1285866	21/11/2012	PRJNA175483	SAMN02469835
Enterococcus pallens	ATCC BAA-351	GCF_000393975.1	Scaffold	358943	5490614	15/05/2013	PRJNA88851	SAMN00809116
Enterococcus haemoperoxidus	ATCC BAA-382	GCF_000393995.1	Scaffold	208343	3580916	15/05/2013	PRJNA88853	SAMN00809117
Enterococcus moraviensis	ATCC BAA-383	GCF_000394015.1	Scaffold	320362	3601841	15/05/2013	PRJNA88855	SAMN00809118
Enterococcus phoeniculicola	ATCC BAA-412	GCF_000394035.1	Scaffold	291579	3931286	15/05/2013	PRJNA88857	SAMN00809120
Enterococcus caccae	ATCC BAA-1240	GCF_000394055.1	Scaffold	240906	3559587	15/05/2013	PRJNA88859	SAMN00809121
Enterococcus dispar	ATCC 51266	GCF_000406945.1	Scaffold	277374	2822228	04/06/2013	PRJNA82531	SAMN02596962
Enterococcus villorum	ATCC 700913	GCF_000407205.1	Scaffold	844450	3060724	04/06/2013	PRJNA191912	SAMN02596958
Enterococcus columbae	ATCC 51263	GCF_000407225.1	Scaffold	235166	2545099	04/06/2013	PRJNA191886	SAMN02596961
Enterococcus avium	ATCC 14025	GCF_000407245.1	Scaffold	652010	4614282	04/06/2013	PRJNA191888	SAMN02596897
Enterococcus saccharolyticus subsp. saccharolyticus	ATCC 43076	GCF_000407285.1	Scaffold	1757281	2604038	04/06/2013	PRJNA191890	SAMN02596896
Enterococcus asini	ATCC 700915	GCF_000407365.1	Contig	2038454	2572706	31/05/2013	PRJNA191897	SAMN02596946
Enterococcus sulfureus	ATCC 49903	GCF_000407605.1	Contig	733717	2301651	31/05/2013	PRJNA191891	SAMN02596964
Bavariicoccus seileri	DSM 19936	GCF_000421665.1	Contig	42553	2242440	09/07/2013	PRJNA188834	SAMN02440893
Tetragenococcus muriaticus	DSM 15685	GCF_000423785.1	Scaffold	30220	2081554	11/07/2013	PRJNA185669	SAMN02441160
Enterococcus crotali	ETRF1	GCF_000633635.1	Contig	408622	3902091	18/04/2014	PRJNA226735	SAMN02729938
Enterococcus massiliensis	AM1	GCF_001050095.1	Contig	503362	2712841	30/06/2015	PRJEB9522	SAMEA3443460
Enterococcus rotai	LMG 26678	GCF_001465345.1	Chromosome	408318	3746111	11/12/2015	PRJNA226735	SAMN04296136
Tetragenococcus solitarius	NBRC 100494	GCF_001544195.1	Contig	48995	2513646	26/01/2016	PRJDB420	SAMD00045733
Enterococcus pseudoavium	NBRC 100491	GCF_001544295.1	Contig	94231	2731874	28/01/2016	PRJDB265	SAMD00045731
Enterococcus canis	NBRC 100695	GCF_001544375.1	Contig	370384	2854214	29/01/2016	PRJDB1349	SAMD00046312
Enterococcus hirae	R17	GCF_001641305.1	Complete Genome	2886481	2960055	10/05/2016	PRJNA319460	SAMN04892752
Tetragenococcus halophilus	MJ4	GCF_001712815.1	Complete Genome	2389470	2389470	25/08/2016	PRJNA289198	SAMN03846930
Enterococcus ureasiticus	DSM 23328	GCF_001730285.1	Contig	508214	3585398	16/09/2016	PRJNA330680	SAMN05420582
Enterococcus plantarum	LMG 26214	GCF_001730295.1	Contig	162885	3135404	16/09/2016	PRJNA330680	SAMN05420586
Enterococcus ureilyticus	LMG 26676	GCF_001730315.1	Contig	217148	3472706	16/09/2016	PRJNA330680	SAMN05420589

Enterococcus quebecensis	LMG 26306	GCF_001730365.1	Contig	361240	3162767	16/09/2016	PRJNA330680	SAMN05420588
Enterococcus rivorum	LMG 25899	GCF_001742285.1	Contig	85487	3806353	22/09/2016	PRJNA330680	SAMN05420592
Enterococcus aquimarinus	DSM 17690	GCF_001885765.1	Contig	145336	2512430	25/11/2016	PRJNA270385	SAMN03267160
Enterococcus hermanniensis	DSM 17122	GCF_001885945.1	Scaffold	267664	2613534	25/11/2016	PRJNA270385	SAMN03267173
Enterococcus italicus	DSM 15952	GCF_001885995.1	Contig	53900	2322207	25/11/2016	PRJNA270385	SAMN03267176
Enterococcus ratti	DSM 15687	GCF_001886195.1	Contig	118830	2485659	25/11/2016	PRJNA270385	SAMN03267184
Enterococcus silesiacus	DSM 22801	GCF_001886225.1	Contig	121840	3915391	25/11/2016	PRJNA270385	SAMN03267185
Enterococcus termitis	DSM 22803	GCF_001886275.1	Scaffold	209758	4127488	25/11/2016	PRJNA270385	SAMN03267188
Enterococcus wangshanyuanii	MN05	GCF_002197645.1	Complete Genome	3842361	4155954	19/06/2017	PRJNA388020	SAMN07171652
Enterococcus canintestini	49	GCF_002269685.1	Scaffold	310733	2734830	24/08/2017	PRJNA292011	SAMN03959821
Enterococcus thailandicus	a523	GCF_002290025.1	Complete Genome	2646250	2646250	12/09/2017	PRJNA400701	SAMN07572979
Enterococcus mundtii	DSM 4838	GCF_002813755.1	Complete Genome	3357452	3504528	07/12/2017	PRJNA353761	SAMN06020033
Enterococcus faecalis	39EA1	GCF_003319815.1	Scaffold	631319	2707177	13/07/2018	PRJNA244756	SAMN02729114
Enterococcus gallinarum	298EA1	GCF_003320875.1	Scaffold	431566	3614506	13/07/2018	PRJNA244756	SAMN02729187
Enterococcus gilvus	CR1	GCF_003343305.1	Complete Genome	2863043	3945324	26/07/2018	PRJNA480543	SAMN09639249
Tetragenococcus osmophilus	JCM 31126	GCF_003795125.1	Complete Genome	2329167	2384293	19/11/2018	PRJNA438374	SAMN08714450
Melissococcus plutonius	DAT561	GCF_003966875.1	Chromo-some	1847807	2067831	24/04/2018	PRJDB6526	SAMD00106497
Enterococcus florum	Gos25-1	GCF_004309355.1	Contig	154984	3994908	15/02/2019	PRJDB8022	SAMD00162630
Enterococcus pingfangensis	241-2-2	GCF_005405205.1	Contig	203181	2017363	29/03/2019	PRJDB7793	SAMD00164306
Enterococcus dongliensis	63-4	GCF_005405225.1	Contig	119838	1814102	29/03/2019	PRJDB7793	SAMD00164307
Enterococcus nangangensis	94-2	GCF_005405245.1	Contig	63973	1993687	29/03/2019	PRJDB7793	SAMD00164308
Enterococcus songbeiensis	85-4	GCF_005405265.1	Contig	118090	2271847	29/03/2019	PRJDB7793	SAMD00164309
Enterococcus viikkiensis	LMG 26075	GCF_005405345.1	Contig	118649	2545311	29/03/2019	PRJDB7793	SAMD00164313
Enterococcus xiangfangensis	11097	GCF_005405365.1	Contig	263199	2652541	29/03/2019	PRJDB7793	SAMD00164314
Tetragenococcus koreensis	NBRC 106072	GCF_007991535.1	Contig	37707	2570246	31/07/2019	PRJDB6218	SAMD00170756
Enterococcus faecium	SRR24	GCF_009734005.1	Complete Genome	2796178	2919198	05/02/2020	PRJNA520784	SAMN10867607
Enterococcus saigonensis	VE80	GCF_011397115.1	Complete Genome	2740356	2844986	26/02/2020	PRJDB9118	SAMD00198474
Enterococcus alcedinis	CCM 8433	GCF_014635985.1	Contig	194119	2686367	11/09/2020	PRJDB10511	SAMD00244887

Enterococcus durans	8L1-82	GCF_015230245.1	Contig	282203	3023465	04/11/2020	PRJNA609650	SAMN14257485
Enterococcus lemanii	DSM 105069	GCF_016909125.1	Contig	52485	2720315	19/02/2021	PRJNA695637	SAMN17620067
Enterococcus hulanensis	DIV0645b	GCF_017316205.1	Scaffold	154879	4730451	10/03/2021	PRJNA313452	SAMN18075564
Enterococcus devriesei	N6	GCF_018917555.1	Contig	328585	3235596	16/06/2021	PRJNA733006	SAMN19374043
Enterococcus raffinosus	F162_2	GCF_019175485.1	Complete Genome	3032004	4255835	08/07/2021	PRJNA719983	SAMN18631319
Enterococcus lactis	CX 2-6_2	GCF_019343125.1	Complete Genome	2559204	2728070	26/07/2021	PRJNA741717	SAMN19908122
Enterococcus timonensis	Marseille-P2817	GCF_900104595.1	Contig	1157085	2123933	27/10/2016	PRJEB16349	SAMEA4519250
Pilibacter termitis	ATCC BAA-1030	GCF_900167335.1	Scaffold	62766	2794241	04/03/2017	PRJEB19562	SAMN02745116
Enterococcus malodoratus	NCTC12365	GCF_900447955.1	Contig	1326400	4628646	01/08/2018	PRJEB6403	SAMEA3714879
Enterococcus cecorum	NCTC12421	GCF_900474605.1	Complete Genome	2421598	2421598	18/06/2018	PRJEB6403	SAMEA3529272
Enterococcus mediterraneensis	Marseille-P4358	GCF_900604485.1	Contig	2402824	2699190	31/10/2018	PRJEB28790	SAMEA4983427
Vagococcus lutrae	LBD1	GCF_000498295.1	Scaffold	132345	1829043	21/11/2013	PRJNA222533	SAMN02597028
Vagococcus teuberi	DSM 21459	GCF_001870205.1	Complete Genome	2169060	2184285	07/11/2016	PRJNA343195	SAMN05781328
Vagococcus penaei	CD276	GCF_001998885.1	Complete Genome	2365304	2365304	14/02/2017	PRJNA362684	SAMN06245872
Vagococcus martis	D7T301	GCF_002026305.1	Contig	2466624	2561923	14/03/2017	PRJNA362683	SAMN06241148
Vagococcus fluvialis	DSM 5731	GCF_003337315.1	Scaffold	184338	2651514	24/07/2018	PRJNA463381	SAMN09074689
Vagococcus sp.	AM17-17	GCF_003471765.1	Scaffold	391219	2258161	06/09/2018	PRJNA482748	SAMN09734843
Vagococcus bubulae	SS1994	GCF_003950315.1	Contig	113661	2499339	17/12/2018	PRJNA359498	SAMN06609019
Vagococcus humatus	JCM 31581	GCF_003950325.1	Contig	408065	2096134	17/12/2018	PRJNA359498	SAMN08717810
Vagococcus vulneris	SS1995	GCF_003950515.1	Contig	121323	2347966	17/12/2018	PRJNA359498	SAMN06609020
Vagococcus salmoninarum	NCFB 2777	GCF_003987495.1	Contig	115236	3104446	17/12/2018	PRJNA359498	SAMN06609018
Vagococcus penaei	LMG 24833	GCF_003987515.1	Contig	124556	2336491	17/12/2018	PRJNA359498	SAMN06609017
Vagococcus lutrae	CCUG 39187	GCF_003987555.1	Contig	141567	1992244	17/12/2018	PRJNA359498	SAMN06609016
Vagococcus fessus	CCUG 41755	GCF_003987565.1	Contig	823406	2257832	17/12/2018	PRJNA359498	SAMN06609014
Vagococcus fluvialis	NCDO 2497	GCF_003987575.1	Contig	184402	2653706	17/12/2018	PRJNA359498	SAMN06609015
Vagococcus entomophilus	DSM 24756	GCF_003987595.1	Contig	1260240	2444868	17/12/2018	PRJNA359498	SAMN06609013
Vagococcus carniphilus	SS1714	GCF_003987635.1	Contig	191814	3080929	17/12/2018	PRJNA359498	SAMN06609011
Vagococcus elongatus	CCUG 51432	GCF_003987645.1	Contig	122341	2879210	17/12/2018	PRJNA359498	SAMN06609012
Vagococcus acidifermentans	LMG 24798	GCF_003987655.1	Contig	155062	2862533	17/12/2018	PRJNA359498	SAMN06609010
Vagococcus sp.	CF-210	GCF_004684045.1	Scaffold	77668	2005002	06/04/2019	PRJNA529778	SAMN11284150

Vagococcus sp.	CF-49	GCF_004792515.1	Complete Genome	1983513	2071287	14/04/2019	PRJNA529772	SAMN11283524
Vagococcus silagei	2B-2	GCF_004795745.1	Scaffold	87525	2525732	16/04/2019	PRJNA516304	SAMN10786376
Vagococcus zengguangii	MN-17	GCF_005145005.1	Complete Genome	2190262	2190262	05/05/2019	PRJNA534179	SAMN11479259
Vagococcus zengguangii	MN-09	GCF_005863195.1	Contig	165950	2041366	22/05/2019	PRJNA543386	SAMN11665394
Vagococcus zengguangii	MN-09	GCF_009676975.1	Contig	165950	2041366	20/11/2019		SAMN12638684
Vagococcus coleopterorum	HDW17A	GCF_011303955.1	Complete Genome	1530734	1530734	16/03/2020	PRJNA609871	SAMN14260687
Vagococcus hydrophili	HDW17B	GCF_011304195.1	Complete Genome	3045783	3045783	16/03/2020	PRJNA609873	SAMN14260688
Vagococcus fluvialis	UFMG-H6	GCF_012102095.1	Scaffold	135292	2679177	06/04/2020	PRJNA615899	SAMN14470522
Vagococcus fluvialis	UFMG-H7	GCF_012102415.1	Scaffold	131353	2993443	06/04/2020	PRJNA615899	SAMN14470523
Vagococcus fluvialis	UFMG-H6B	GCF_012102505.1	Scaffold	112454	2850525	06/04/2020	PRJNA435944	SAMN14486420
Vagococcus lutrae	DS2263-02	GCF_012396665.1	Contig	84408	1877641	15/04/2020	PRJNA622446	SAMN14517839
Vagococcus carniphilus	ATCC BAA-640	GCF_014397115.1	Complete Genome	3020833	3128613	02/09/2020	PRJNA658884	SAMN15892503
Vagococcus salmoninarum	VS-1	GCF_015209575.1	Scaffold	80381	3060919	02/11/2020	PRJNA670681	SAMN16516787
Vagococcus lutrae	8B3	GCF_016760265.1	Contig	63312	2026740	14/01/2021	PRJDB8437	SAMD00175810
Vagococcus lutrae	9A8	GCF_016760285.1	Contig	69508	2027140	14/01/2021	PRJDB8438	SAMD00175811
Vagococcus lutrae	9B9	GCF_016760305.1	Contig	69508	2011496	14/01/2021	PRJDB8439	SAMD00175812
Vagococcus fluvialis	DIV0098	GCF_017315815.1	Scaffold	152434	2675919	10/03/2021	PRJNA313452	SAMN18075576
Vagococcus fluvialis	DIV0657d	GCF_017315855.1	Scaffold	68124	2742024	10/03/2021	PRJNA313452	SAMN18075578
Vagococcus fluvialis	DIV0648b	GCF_017315875.1	Scaffold	380315	3127155	10/03/2021	PRJNA313452	SAMN18075577
Vagococcus fluvialis	DIV0068	GCF_017315905.1	Scaffold	61865	2742473	10/03/2021	PRJNA313452	SAMN18075575
Vagococcus fluvialis	DIV0038b	GCF_017315915.1	Contig	95556	2832535	10/03/2021	PRJNA313452	SAMN18075574
Vagococcus fluvialis	DIV0015	GCF_017315965.1	Scaffold	365590	2876592	10/03/2021	PRJNA313452	SAMN18075573
Vagococcus fluvialis	MSG3302	GCF_017316045.1	Contig	228958	2760670	10/03/2021	PRJNA313452	SAMN18075579
Vagococcus sp.	DIV0080	GCF_017316185.1	Scaffold	93803	2728262	10/03/2021	PRJNA313452	SAMN09399635
Vagococcus sp.	BWB3-3	GCF_017829975.1	Scaffold	236989	5705793	07/04/2021	PRJNA682501	SAMN16992987
Vagococcus lutrae	BN31	GCF_019797785.1	Complete Genome	2030321	2083250	30/08/2021	PRJNA756377	SAMN20864220
Vagococcus fluvialis	bH819	GCF_900163795.1	Contig	319188	2950994	29/04/2017	PRJEB19180	SAMEA81165418
Vagococcus teuberi	MGYG-HGUT-00234	GCF_902364615.1	Scaffold	391219	2258161	22/08/2019	PRJEB33885	SAMEA5849736

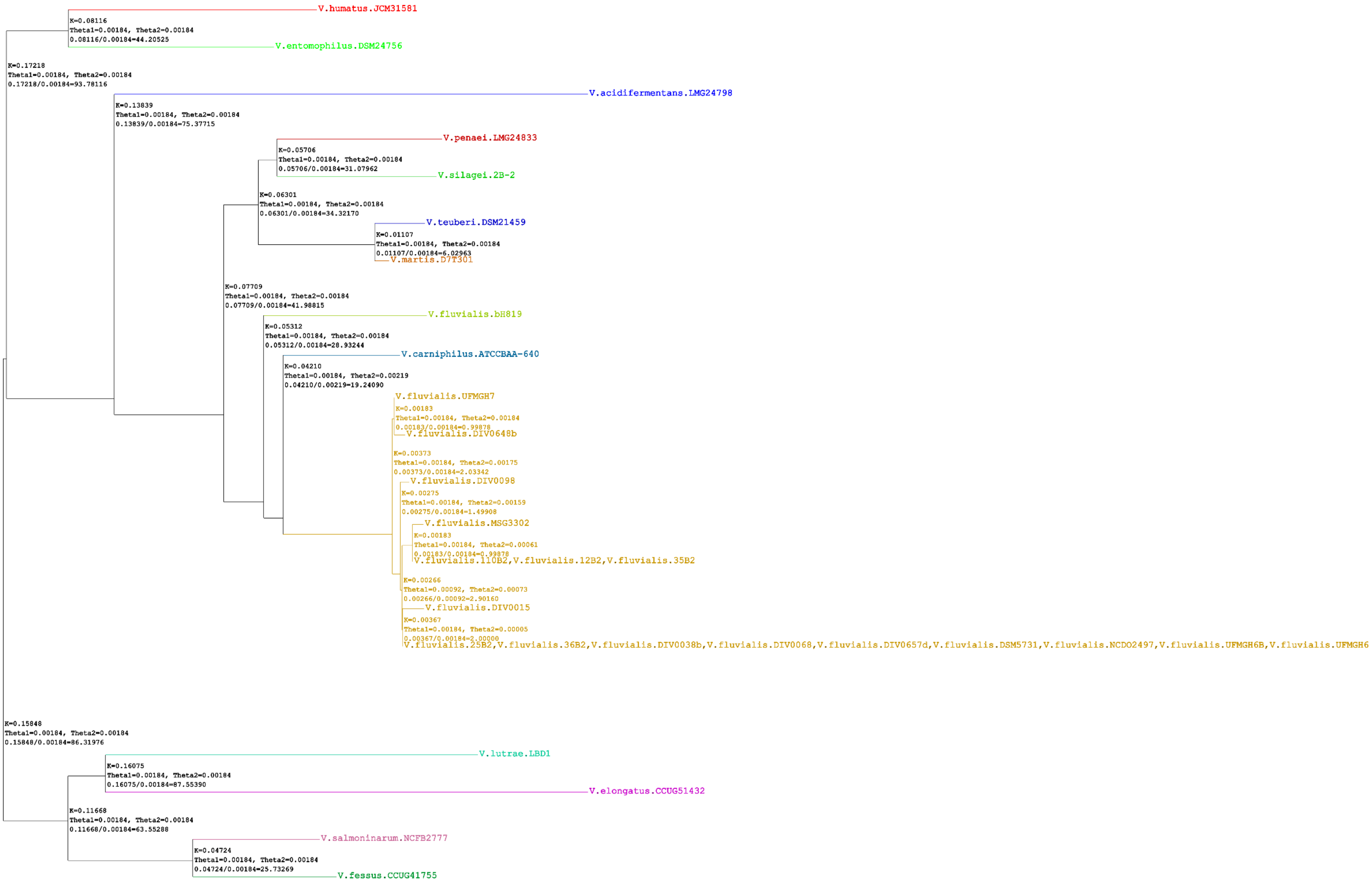

Supplementary Figure S10. KoT species delimitation of the *V. fluvialis* genomes and the representative genomes of other *Vagococcus* species.

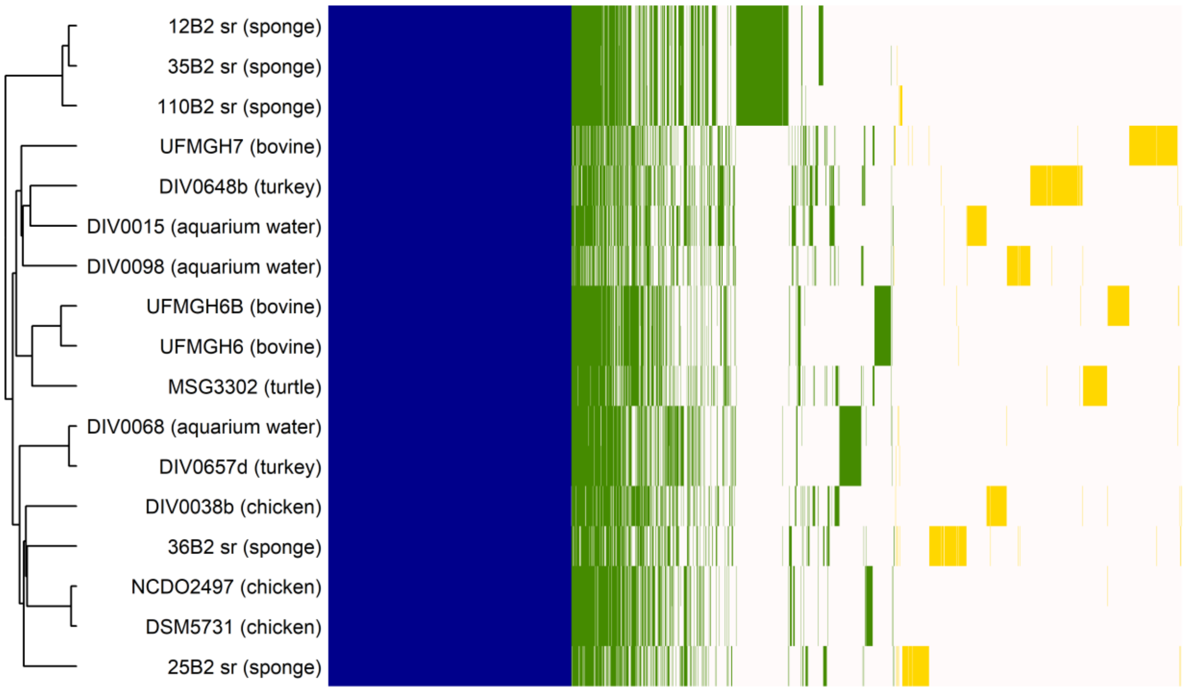

Supplementary Figure S11. Gene presence/absence matrix of all orthologous gene clusters in the *V. fluvialis* pan-genome, using the short-read assemblies of the sponge-isolated *V. fluvialis* strains genomes (sr). The genomes are ordered according to the dendrogram constructed using hierarchical clustering based on the gene presence/absence matrix. Genes shown in blue represent the core genome (present in all *V. fluvialis* genomes), those in green represent the shell genomes (present in more than 2 genomes), and those in yellow are unique genes (present in only one genome).
